# Anthraquinone and its derivatives as sustainable materials for electrochemical applications – a joint experimental and theoretical investigation of the redox potential in solution†

**DOI:** 10.1039/d2cp01717b

**Published:** 2022-06-01

**Authors:** Josef M. Gallmetzer, Stefanie Kröll, Daniel Werner, Dominik Wielend, Mihai Irimia-Vladu, Engelbert Portenkirchner, Niyazi Serdar Sariciftci, Thomas S. Hofer

**Affiliations:** Theoretical Chemistry, Division, Institute of General, Inorganic and Theoretical Chemistry, Center for Chemistry and Biomedicine, University of Innsbruck Innrain 80-82 A-6020 Innsbruck Austria t.hofer@uibk.ac.at; Institute of Physical Chemistry, Josef-Möller-Haus, University of Innsbruck Innrain 52c A-6020, Innsbruck Austria engelbert.portenkirchner@uibk.ac; Linz Institute for Organic Solar Cells (LIOS), Institute of Physical Chemistry, Johannes Kepler University Linz Altenberger Strasse 69 4040 Linz Austria

## Abstract

Anthraquinone (AQ) has long been identified as a highly promising lead structure for various applications in organic electronics. Considering the enormous number of possible substitution patterns of the AQ lead structure, with only a minority being commercially available, a systematic experimental screening of the associated electrochemical potentials represents a highly challenging and time consuming task, which can be greatly enhanced *via* suitable virtual pre-screening techniques. In this work the calculated electrochemical reduction potentials of pristine AQ and 12 hydroxy- or/and amino-substituted AQ derivatives in *N*,*N*-dimethylformamide have been correlated against newly measured experimental data. In addition to the calculations performed using density functional theory (DFT), the performance of different semi-empirical density functional tight binding (DFTB) approaches has been critically assessed. It was shown that the SCC DFTB/3ob parametrization in conjunction with the COSMO solvation model provides a highly adequate description of the electrochemical potentials also in the case of the two-fold reduced species. While the quality in the correlation against the experimental data proved to be slightly inferior compared to the employed DFT approach, the highly advantageous cost-accuracy ratio of the SCC DFTB/3ob/COSMO framework has important implications in the formulation of hierarchical screening strategies for materials associated with organic electronics. Based on the observed performance, the low-cost method provides sufficiently accurate results to execute efficient pre-screening protocols, which may then be followed by a DFT-based refinement of the best candidate structures to facilitate a systematic search for new, high-performance organic electronic materials.

## Abstract 2

Correlations between quantum chemically determined 1- and 2e^−^ electrochemical potentials for different AQ systems obtained at DFT and DFTB levels show very good agreement with newly measured experimental data determined *via* CV measurements.

## Graphical abstract

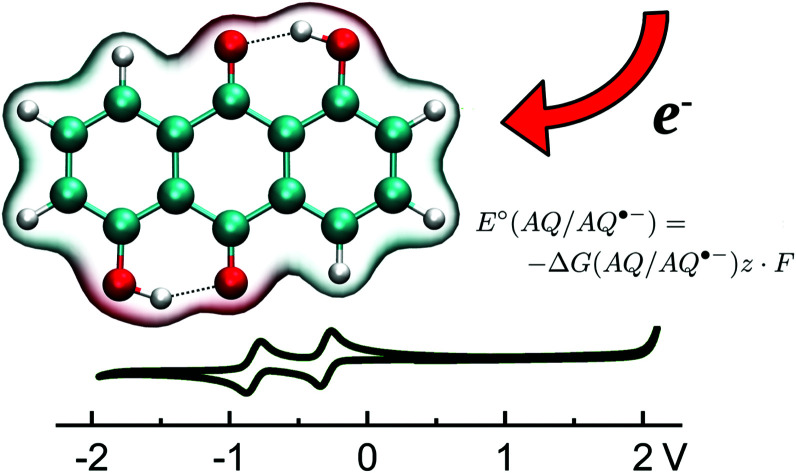

## 1 Introduction

Organic materials in the form of small molecules and polymers have been recently implemented in electronic applications like organic field effect transistors (OFETs) as indispensable components of integrated circuits and sensors, as well as in organic light emitting diodes (OLEDs) as electric displays and plastic solar cells.^1–3^ With the huge performance progress accomplished in the case of the semiconducting layer over the past two decades,^4,5^ there is a clear trend of departing from inorganic in favour of organic components. Additionally, there is tremendous energy expended in the fabrication process of high performance inorganic semiconductor materials.^6^ Another huge benefit of small organic molecules is their capability of interaction with biological systems, enabling electrochemical sensing of DNA,^7,8^ saccharides^9^ and even ionic compounds such as F^−^, CN^−^ and HSO_4_^−^ in cancer cell imaging.^10^

There are basically two main advantages of this development: the first one is the high formability and flexibility of organic materials and polymer thin films thereof compared to their inorganic analogs that are mostly brittle.^11–13^ The second key factor is the possibility of fabricating devices that can be integrated into controllable biodegradation routes and hence achieve environmental sustainability in electronics development.^14–17^

As part of this abovementioned thrust, organic electrode materials have received increasing interest in the field of organic electronics for two main reasons. On the one hand, organic materials are more environmentally friendly than transition metal oxides, since they can be synthesized from biomass and are consequently biodegradable, combined with relatively low costs.^18,19^ On the other hand, the use of organic electrode materials enables the formulation of Na-ion batteries.^20,21^ At present, the development of Na-ion batteries is limited by the inability of Na to form stable graphite intercalation compounds under moderate conditions.^22^ Organic materials, however, have a more open and porous structure and are not as sensitive to the Na-ion radius and its related formation energies.^23^ Consequently, organic materials are promising candidates for use in Na-ion batteries.^24^

Among the various types of organic materials, small molecular compounds are considered as highly promising candidates. In particular, quinone-based derivatives, such as anthraquinones (AQ),^25–27^ are intensely studied for their potential application as a cathode material in metal–ion batteries.^28^ This is *inter alia* a consequence of their capability to accommodate a two-electron reduction, resulting in a high energy storage capability in combination with fast charging/discharging rates.^29,30^ One key parameter defining the energy storage capability of a battery is its associated cell voltage. The latter is dependent on the redox potentials of both, the active cathode and anode materials. The redox potential of AQ, and other organic carbonyl compounds, can be tailored by the introduction of electron-donating or -withdrawing groups, resulting in an increase or decrease of the respective electrochemical potentials.^31–34^ This tunability of potential is a powerful tool not only to increase the respective energy density in a battery application, but also to mitigate problems related to interface instability, safety issues and stability limits associated with most organic electronic devices.^35,36^ In addition, AQ and its derivatives have shown great promise as an electrode material in the electrochemical capture and release of CO_2_.^37,38^

**Fig. 1:**
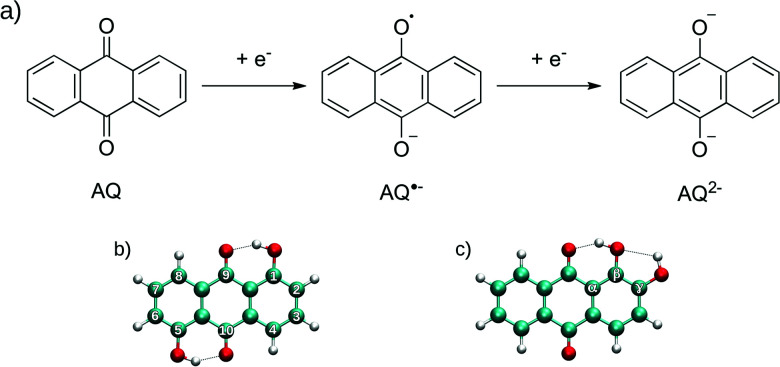
(a) Overview of the individual electrochemical reaction steps taking the example of AQ. (b and c) Numbering convention and relative positioning with respect to the carbonyl groups taking the example of 1,5- (b) and 1,2-OH-AQ (c), respectively (minimum structures obtained at the B3LYP+D3/6-31++G(d,p)/SMD level, visualization *via* VMD^87^).

Virtual screening approaches based on quantum mechanical (QM) calculation methods provide an alternative and highly promising route to pre-assess potential candidate structures. Considering the numerous possible substitutions of the AQ lead structure (see Fig. 1), a large number of potential derivatives can be formulated with only a minority of those compounds being commercially available. Thus, a systematic experimental probing of AQ-derivatives focusing on their potential application in organic electronic devices (such as batteries or transistors) is *inter alia* limited by the requirement to synthesize non-available compounds, often followed by intricate protocols for purification.

Huskinson *et al.* employed density functional theory (DFT) calculations to investigate the change in the reduction potential of 9,10-anthraquinone-2,7-disulphonic acid (AQDS) derivatives as a function of the number of hydroxy groups.^39^ Similarly, Er and coworkers reported a systematic DFT screening of benzo- (BQ), naphtho- (NQ) and anthraquinone derivatives, focusing on their potential use in redox flow batteries.^40^ Based on an extensive combinatorial library of more than 10 000 virtual conformers, key properties such as the reduction potential and the solvation free energy were estimated. Huynh *et al.* have investigated the 1e^−^ and 2e^−^ reduction potentials of 134 different quinone derivatives employing DFT calculations within an isodesmic reaction framework.^41^ In this approach, the redox potentials of individual compounds have been determined relative to the reduction reactions of 1,4-benzoquinone acting as reference species. In addition, a total of 15 BQ-derivatives have been measured experimentally acting as the validation set. Although an overall linear correlation was identified, deviations from linearity were observed for a number of compounds. Moreover, the data show that BQ does not appear to be an ideal reference when aiming at calculations of NQ and AQ derivatives. A recent study by Schwan and coworkers^42^ also focused on a DFT-based virtual screening of several BQ, NQ and AQ compounds for potential applications in quinone-based redox-flow systems. Based on the obtained results the impact of the nature and position of substituents of the respective compounds was characterized.

These studies clearly highlight the capabilities of high throughput quantum chemical screening strategies in the area of materials development. Typically, DFT approaches^43,44^ at the generalized gradient approximation (GGA) and hybrid levels represent the *de facto* standard in these investigations, as they provide an acceptable compromise between accuracy of results and computational demand. Nevertheless, DFT methods are known to scale unfavourably with the system size, which may ultimately prove to be a limiting factor in this kind of screening approach.

A widely employed alternative to DFT and *ab initio* calculations are semi-empirical QM approaches.^45,46^ These methods are based on a number of approximations introduced in DFT or/and Hartree–Fock theory, thereby sacrificing accuracy in favour of more efficient execution times. A particularly successful class of semi-empirical QM methods are density functional tight binding (DFTB) approaches.^47–51^ In this framework the DFT Kohn–Sham energy of the chemical system^43,44^ is expressed *via* a Taylor series with respect to variations in the equilibrium density. It was shown that the resulting expression is highly similar to the comparably simple Hamiltonians formulated in tight binding (TB) theory.^52^ In particular, the self-consistent charge density functional tight binding (SCC DFTB) approach^53,54^ was shown to display an exceptional accuracy/effort ratio in calculations of a broad range of chemical systems, provided the investigated systems remain within the scope of the applied parametrization. This leaves DFTB approaches to some extent susceptible to methodical shortcomings, since the transferability of a particular parametrization to a different class of chemical systems is not always feasible.

In the context of the current investigation focusing on the electrochemical potential of different AQ derivatives, the increasing negative charge upon subsequent reduction of the target molecules might prove to be a limiting factor in the application of DFTB-based approaches. On the other hand, the identification of an adequate parametrization capable of providing accurate estimates for the electrochemical potentials can be expected to greatly enhance the scope and applicability of virtual screening approaches focused on organic electrode materials.

In this work a hierarchical computation study employing both DFT and SCC DFTB approaches for the estimation of the electrochemical potential of AQ and 12 AQ-derivatives has been carried out, which are benchmarked against newly determined experimental data obtained *via* cyclic voltammetry (CV) measurements. In addition to focusing on the comparison of the calculated data against the experimental reference, correlations between the employed theoretical methods provide detailed insight into the strengths and weaknesses of the individual approaches.

## 2 Methods

Details on the experimental and theoretical determination of the electrochemical potential for pristine AQ and 12 AQ-derivatives are outlined. The individual steps in the electrochemical reaction of AQ are depicted in Fig. 1 along with an overview of the commonly employed numbering convention and relative positioning indices.

### 2.1 Target systems

The AQ-based molecules investigated in this work were selected in order to provide a thorough study of the following commercially available mono- and di-substituted AQ derivatives carrying amino (NH_2_) and hydroxy (OH) substituents: 1-OH-AQ, 2-OH-AQ, 1-NH_2_-AQ, 2-NH_2_-AQ, 1,2-OH-AQ, 1,4-OH-AQ, 1,5-OH-AQ, 1,8-OH-AQ, 1,4-NH_2_-AQ, 1,2-NH_2_-AQ, 2,6-NH_2_-AQ and 1-NH_2_-4-OH-AQ.

### 2.2 Sample preparation and purification

Each of the 13 target molecules investigated in this work was purified from the as-received material (chemical suppliers TCI Europe and Sigma-Aldrich) *via* the train sublimation method. The procedure was accomplished in a vacuum at a pressure below 1 × 10^−5^ mbar, by using a quartz and two borosilicate glass tubes that were fused by flame and inserted into the carrier quartz tube. The borosilicate tubes had a double purpose: they served as a confinement of the source material and as a means of recovering the sublimed material at the completion of the sublimation process. Each material investigated here typically sublimed within a 24 hour period at a particular temperature ranging from 150 °C to 200 °C. It should be noted that each organic material was purified twice in order to increase its purity for subsequent experimental work. The importance of having high quality organic materials was thoroughly analyzed in a previous publication.^55^

### 2.3 Experimental measurements

Cyclic voltammetry (CV) experiments were performed on an IPS-Jaissle potentiostat/galvanostat PGU10V-100mA inside a glove box under an inert nitrogen (N_2_) atmosphere. The electrochemical cell consisted of a one-compartment cell employing a 2 mm platinum (Pt) disc-type working electrode (PalmSens), a platinum counter electrode and a quasi-reference Ag/AgCl electrode. The target material investigated was weighed into the electrochemical cell outside the glove box in a way to achieve a final concentration of 2 mM or 5 mM (depending on the available quantity of the respective sample) in overall 10 mL electrolyte solution. Inside the glove box, 10 mL electrolyte solution consisting of 0.1 M tetrabutylammonium hexafluorophosphate (TBAPF_6_, >99.0%, Sigma Aldrich) in *N*,*N*-dimethyl formamide (DMF, >99.8%, Sigma Aldrich) was added and dissolved while stirring for 30 min. The CV scans were performed at a scan rate of 200 mV s^−1^ for all materials. For conversion of the applied potential *versus* the standard hydrogen electrode (SHE), after each measurement, a calibration with ferrocene (98%, Sigma Aldrich, *E*_1/2_ of Fc/Fc^+^ with 640 mV *vs.* SHE^56^) was carried out.

### 2.4 Calculation settings

To investigate the reduction potentials of the different AQ systems density functional theory (DFT) calculations at the B3LYP^57^/6-31++G(d,p)^58–61^ level have been carried out using Gaussian16,^62^ applying also the GD3BJ dispersion correction of Grimme and coworkers.^63^ The structures were optimized in implicit *N*,*N*-dimethylformamide (DMF, relative permittivity *ε* = 37.219^62^) using the solvation model density (SMD) approach.^64^

All systems were also investigated using self-consistent charge density functional tight binding (SCC DFTB)^65,66^ employing the 3ob^67^ as well as the mio^68^ parameter set in conjunction with the DFT-D4 dispersion correction.^69–71^ Solvent effects for DMF were taken into account using the implicit conductor-like screening model (COSMO)^72,73^ (relative permittivity *ε* = 37.0, molar mass *M* = 73.1 g mol^−1^ and density *ρ* = 0.95 kg L^−1^). The SCC DFTB calculations were performed using the open-source program dftbplus (v21.1).^74^

In addition, calculations employing the second-generation extended tight binding method for geometries, frequencies and non-bonded interactions (GFN2-xTB)^75–78^ were carried out using the open-source program *x*TB of Grimme and coworkers.^78^ In this case, two implicit solvation models are available, namely the analytical linearized Poisson–Boltzmann (ALPB) method^79^ and the generalized Born model with surface area contributions (GBSA).^79^ The respective parameters as reported by Ehlert and coworkers have been applied.^79^

The target systems have been subject to energy minimization followed by a frequency calculation *via* a respective Hessian analysis. All eigenvalues of the latter were confirmed to be positive for each system, thereby not considering eigenvalues associated with invariant degrees of freedom. Based on the Hessian data a thermochemical analysis under standard conditions *T* = 298.15 K and *p* = 1.013 bar was carried out to estimate the associated free energy *G*, taking also the respective zero-point energy, entropic contributions and solvation effects into account.^80^ This procedure was repeated for all levels of theory considered in this work.

### 2.5 Calculation of reduction potentials

The calculation of the electrochemical potentials for the two reduction steps was performed by calculating the respective Gibbs free energy *G* including solvation effects. The first reduction step (AQ/AQ˙^−^) consists of the reaction starting from neutral AQ and resulting in the radical anion AQ˙^−^. The associated reaction Gibbs free energy Δ*G*, was calculated according to Huynh *et al.*^41^ as

1 Δ*G*(AQ/AQ˙^−^) = *G*(AQ˙^−^) − *G*(AQ),

followed by the calculation of the reduction potential *via*2
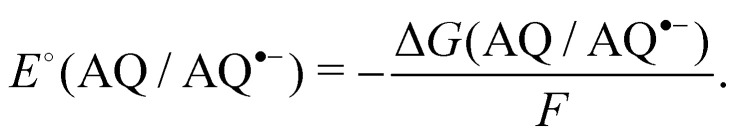
In the case of the second reduction step (AQ/AQ^2−^) the average over the individual half reactions^41^ given as3
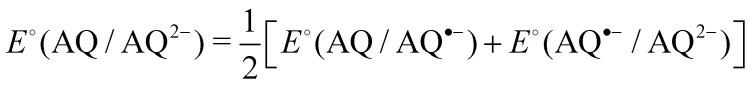
was determined. The individual contributions correspond to the potentials obtained for the first and second reduction step, respectively.

### 2.6 Isodesmic reduction reaction

All measured reduction potentials have been determined relative to the predefined reference potential of the standard hydrogen electrode (SHE), and consequently, all calculation data should be provided relative to the SHE as well. Therefore, the theoretically determined potentials of AQ were set as reference for the calculated AQ derivatives. For all considered systems the reduction potentials against pure AQ were then evaluated as follows

4 Δ*E*°(X–AQ/X–AQ˙^−^) = Δ*E*°(AQ/AQ˙^−^) + [*E*°(X–AQ/X–AQ˙^−^) − *E*°(AQ/AQ˙^−^)],

thus providing the respective electrochemical potential Δ*E*°(X–AQ/X–AQ˙^−^) relative to the selected reference molecule,^41^ enabling direct comparison with the experimentally determined data. It should be noted that the employed reference potential used for pristine AQ was not taken from the literature. Instead, a newly measured value obtained under identical conditions as applied in the measurements of the target systems was utilized.

The methodology provides the electrochemical potential of the target molecule X–AQ relative to the selected reference molecule, in this case the unsubstituted AQ. The reason for such an approach is that the free energy associated with the transferred electron cancels on the educt and product side of the reaction (see also Table S12, ESI†). The disadvantage of this method is that the redox potential of the reference molecule must be obtained from another source (usually experimental measurements). Therefore, it is only possible to predict electrochemical potential data relative to those of the reference molecule. In the work of Huynh *et al.*,^41^ pure benzoquinone (BQ) was used as a reference, which provided data for various BQ derivatives in very good agreement with experimentally measured reference values. However, it was observed that BQ is not an ideal reference molecule for the calculation of naphtho- and anthraquinone derivatives.

The calculation procedure outlined above was then applied in the case of the second reduction step as well.

### 2.7 Molecular properties from DFT calculation data

To investigate the molecular properties based on the orbital energies of different neutral AQ systems, the DFT calculations at the B3LYP+D3/6-31++G(d,p) level in implicit solvation were further analyzed. According to Zhan *et al.*,^81^ the ionization potential (IP) and electron affinity (EA) can be approximately derived from the HOMO and LUMO orbital energies, provided the basis set is sufficiently large to obtain a negative LUMO energy. Although they do not represent the exact absolute value for IP and EA, the correlation can provide suitable values due to the qualitative trend of the two values, which were shown to display an excellent linear relationship.^81^5
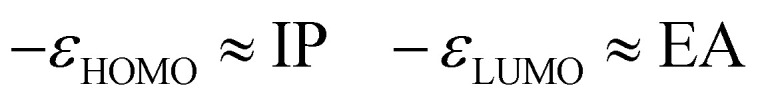
Furthermore, IP and EA data enable the estimations of the electron affinity *χ* and the hardness *η*, respectively.6
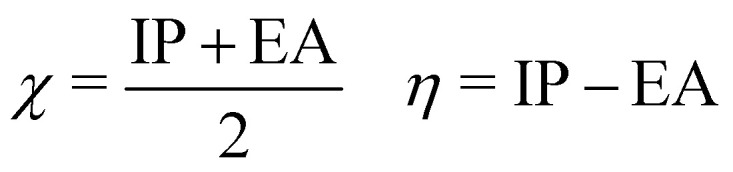
While it is difficult to obtain such key properties of molecular species by experimental means, they can be derived directly from electronic structure calculation data, such as the HOMO and LUMO energies. Since due to different approximations the latter are not accurately represented in the DFTB approach, this analysis was performed only for the most accurate method, namely DFT at the B3LYP+D3/6-31++G(d,p)/SMD level.

In addition, electrostatic surface potentials (ESPs)^26,82^ were generated for all AQ derivatives, considering again only the most accurate calculation method being DFT at the B3LYP+D3/6-31++G(d,p)/SMD level, and the compounds were sorted according to their respective measured electrochemical potentials.

## 3 Results and discussion

### 3.1 Results of the CV measurements

**Fig. 2:**
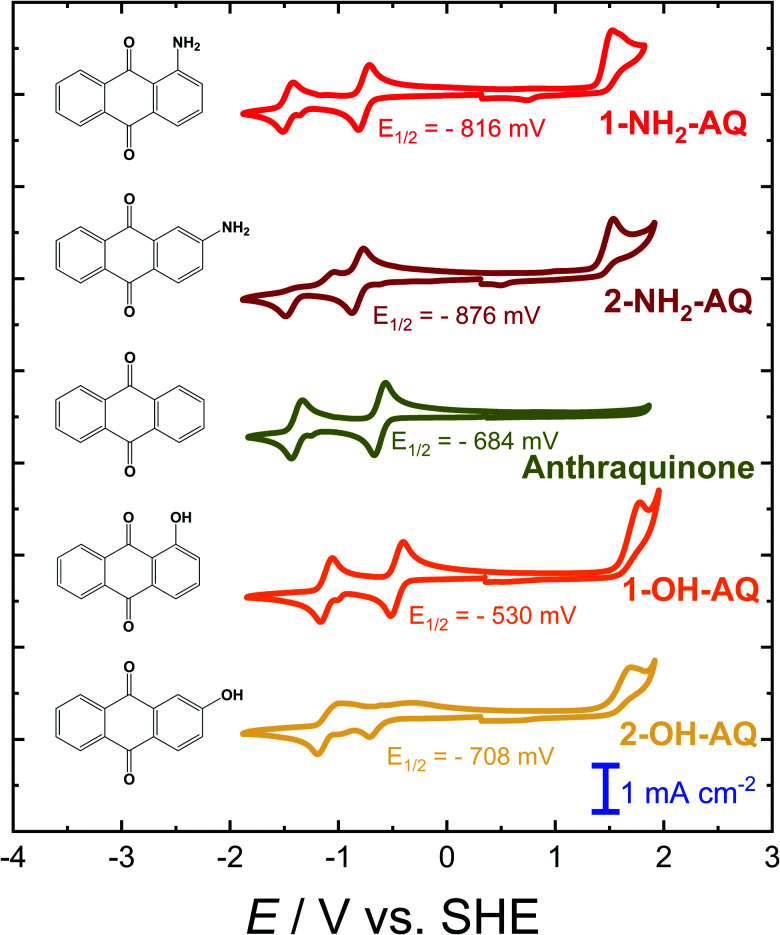
CV graphs of 2 mM solutions of AQ and its mono-substituted hydroxy- and amino-derivatives in 0.1 M TBAPF_6_/DMF solution at a scan rate of 200 mV s^−1^. The given *E*_1/2_ potentials refer to the first reduction feature.

A comparison of the CV graphs obtained for pristine AQ and its mono-substituted derivatives is depicted in Fig. 2. In all cases the CV curves display two separated one-electron reduction peaks with associated re-oxidation features. In the case of unsubstituted AQ textbook-like reversible reduction features with a potential of −684 mV were observed, while no electrochemically accessible oxidation features were present.^83^ Substitutions by an amino (NH_2_) group, having a positive mesomeric effect, are known to lead to an increase in the electron density within the aromatic system. This is reflected by characteristic cathodic shifts of the reduction potential. As can be seen from the respective CV plots in Fig. 2, amino-substitution in the γ-position results in a stronger cathodic shift compared to its β-counterpart. However, it should be noted that in the case of 2-NH_2_-AQ, the second reduction feature is significantly less reversible. In addition to the effect on the reduction behavior, the increase in electron density facilitates oxidation reactions which can be expected to occur at the nitrogen atom, resulting in the formation of an iminium ion.

Although hydroxy (OH) substitution is also expected to display the same positive mesomeric effect, only in the case of 2-OH-AQ a slight cathodic shift of the first reduction potential is observed in the CV graph (see Fig. 2). On the other hand, 1-OH-AQ was determined to possess a reduction potential shifted anodically by 154 mV. This positive shift in the reduction potential for the OH-substitution in the β-position is in general already well described in the literature, from both an experimental and theoretical point of view. It is attributed to the intramolecular stabilization of the radical AQ species by adjacent hydroxy groups.^31,84^ Recently, it was concluded that this anodic shift can be directly traced to the associated hydrogen bonding, as it was only observed for OH-groups, whereas a cathodic shift of the reduction potential was identified for alkoxy substituents^85^ (see also examples in Fig. 1b and c). As a consequence of the less-favorable γ-position of the hydroxy group in 2-OH-AQ, this stabilization effect cannot be observed. According to the literature, substitution effects in the γ-position are in general less pronounced and more diverse.^31^

Overall, it should be noted that the CV characteristics of 2-OH-AQ is markedly different from an ideal electrochemical response. While the reduction peaks show the expected, well-separated, two-fold one-electron reduction peaks, the re-oxidation features are not well resolved. Moreover, the re-oxidation waves are significantly broadened, and the oxidation peak currents are strongly reduced compared to those observed during reduction. Additionally, there appears to be a large separation in peak potentials between the reduction and re-oxidation peaks of approximately 110 mV, indicating the presence of significant overpotentials for the back oxidation reactions. One possible explanation for this could be the formation of dimers following the first electron reduction reaction to the radical anion. Dimerization reactions of AQs substituted by disulfonic acid in the 2-position have been previously reported.^25,86^ Based on this assessment it can be expected that 2-OH-AQ might prove to be a particularly challenging system in virtual screening, since effects such as dimerization and other side reactions cannot be adequately represented if only a single molecule is considered in the quantum chemical calculations.

**Fig. 3:**
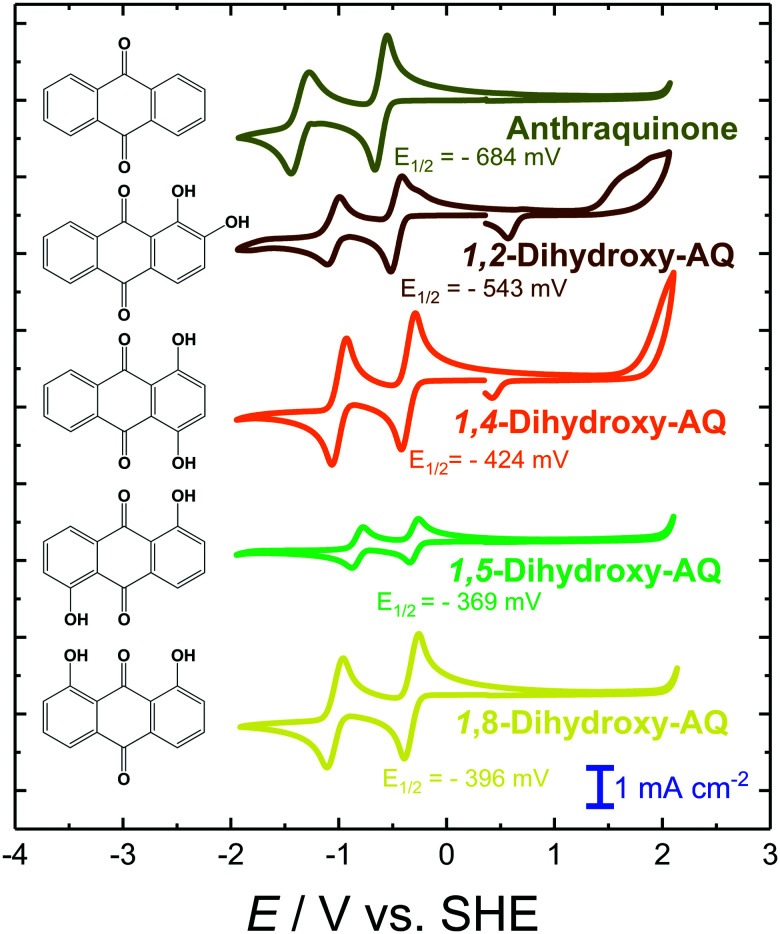
CV graphs of 5 mM solutions of AQ and its di-substituted hydroxy-derivatives in 0.1 M TBAPF_6_/DMF solution at a scan rate of 200 mV s^−1^. The given *E*_1/2_ potentials refer to the first reduction feature.

In order to extend the number of amino- and hydroxy-derivatives, di-substituted –OH and –NH_2_ analogues were also investigated. In Fig. 3 the CV graphs of different dihydroxy-AQ systems are compared. Similar to that observed in the case of a single OH-group in the β-position, this trend is continued if a second OH-group is placed at an equivalent position of the AQ lead structure, *i.e.* positions 4, 5 or 8. Comparing the reduction potentials of these β-only derivatives, 1,4-, 1,5- and 1,8-OH-AQ, revealed a positive shift in the reduction potentials. This result demonstrates the importance of the stabilization of both reduced carbonyl groups along with the distribution of the OH substituents in the AQ host scaffold. These findings are in good agreement with the findings of Ashnagar *et al.*^84^ In the case of 1,2-OH-AQ a slight cathodic shift in the first reduction potential by 13 mV, compared to 1-OH-AQ, is observed. Moreover, this molecule is the only OH-AQ derivative exhibiting a clear oxidation peak.

**Fig. 4:**
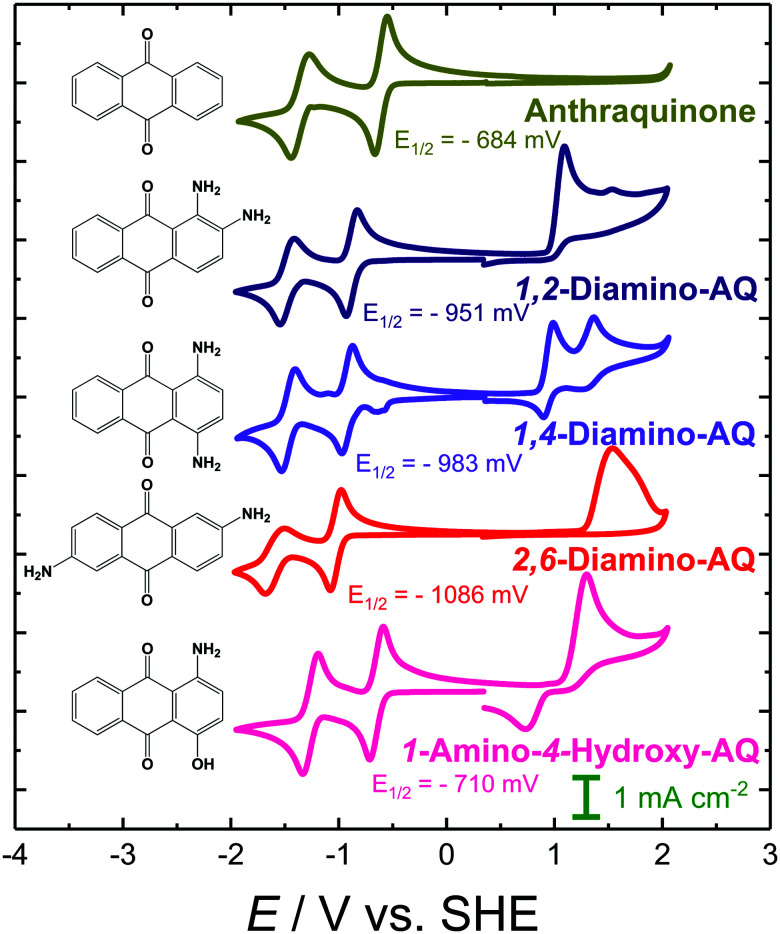
CV graphs of 5 mM solutions of AQ, its di-substituted amino-derivatives and the mixed derivative 1-OH-4-NH_2_-AQ in 0.1 M TBAPF_6_/DMF solution at a scan rate of 200 mV s^−1^. The given *E*_1/2_ potentials refer to the first reduction feature.

The CV plots of the doubly substituted amino-derivatives are depicted in Fig. 4. They reveal the expected opposing effect of the substitution positions with 2,6-NH_2_-AQ, displaying the strongest, cathodic shift of the di-amino materials investigated, while the most positive reduction potential was observed in the case of 1,2-NH_2_-AQ. For the oxidation reaction, amino groups in the β-position appear to be essential for reversibility. In particular, 1,4-NH_2_-AQ shows two reversible oxidation peaks, 1,2-NH_2_-AQ two quasi-reversible oxidation features and 2,6-NH_2_-AQ only a single irreversible oxidation peak. These results can be explained by the ideal charge delocalization attributed to amino groups in the *para*-position of an aromatic moiety, which is structurally similar to the reduction of *para*-quinone groups.

Finally, the 1-NH_2_-4-OH-AQ system, also included in Fig. 4, is of particular interest, since it is the only investigated molecule carrying two different substituents in the β-positions. Interestingly, the opposing trends of the two functional groups nearly cancel, resulting only in a slight cathodic shift in the reduction potential by 26 mV. In the series of amino-substituted AQ derivatives, 1-NH_2_-4-OH-AQ displays an oxidation feature, which can be attributed to the presence of the amino group, since the investigated dihydroxy derivatives did not show pronounced oxidation features at such moderate potentials.

In summary, the different substitution patterns applied to the AQ lead structure exhibit a versatile tuneability in the associated reduction potentials. The observed difference of more than 700 mV between 1,5-OH-AQ and 2,6-NH_2_-AQ paves the ground for the versatile use of AQ derivatives in a number of organic electronic applications.

### 3.2 Correlation between the experiment and theory

All experimentally investigated AQ derivatives have been subject to quantum chemical calculations at different levels of theory. Based on the resulting estimates for the associated free energies *G*, the reduction potentials for the different AQ derivatives have been determined by an isodesmic reaction framework with unsubstituted AQ employed as reference. In order to assess the accuracy of the theoretical predictions, the resulting electrochemical potentials have been compared to the newly measured experimental data.

**Fig. 5:**
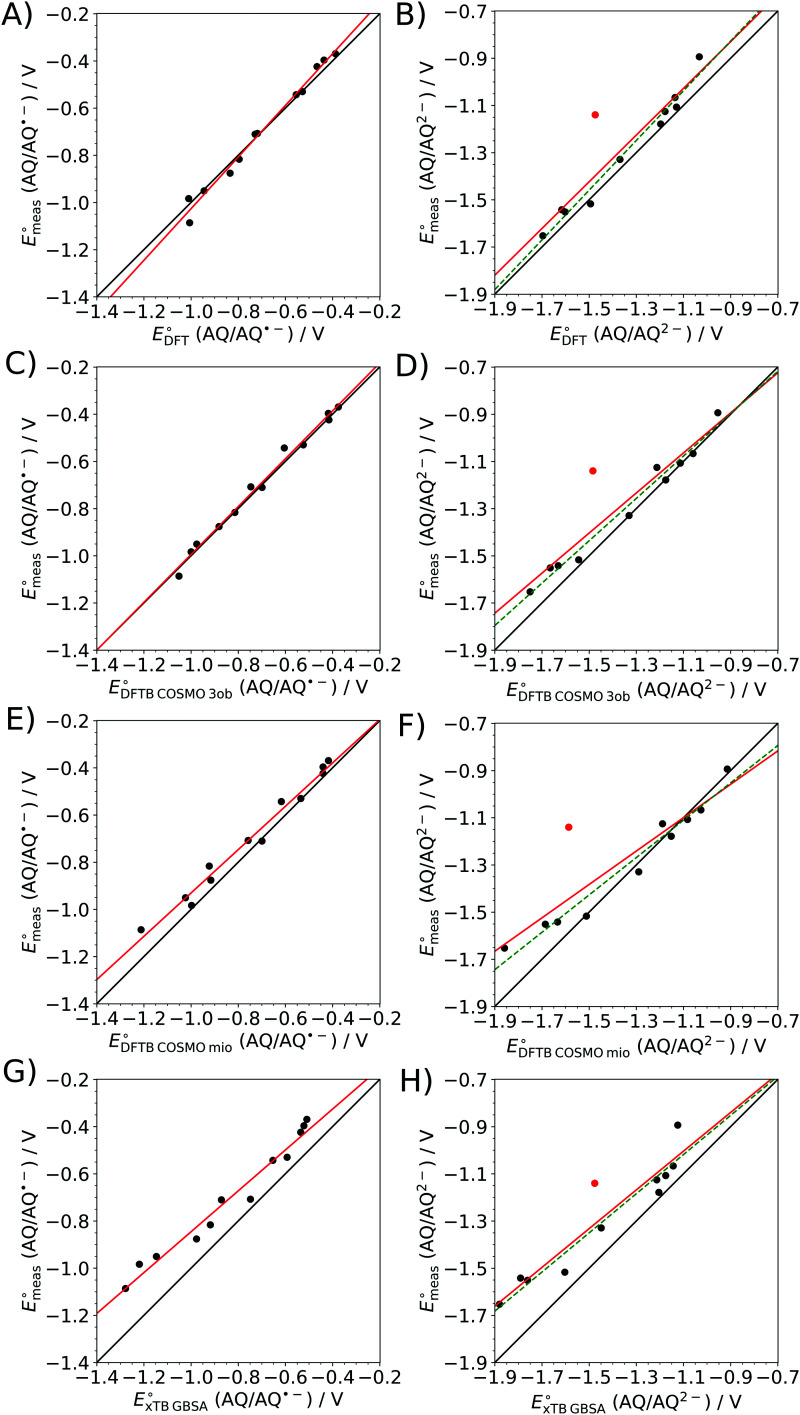
Correlation between the experimentally and theoretically determined electrochemical potentials for the first (left) and second (right) reduction step of the considered AQ-derivatives obtained at (A and B) B3LYP+D3/6-31++G(d,p), (C and D) SCC DFTB/3ob, (E and F) SCC DFTB/mio and (G and H) GFN2-xTB/GBSA levels in implicit solvation. Black line: ideal correlation. Red line: linear regression. Green dashed line: linear regression without the outlier 2-OH-AQ marked by a red dot.

The associated correlation plots are depicted in Fig. 5 and were determined *via* linear fits of the form

7 *y* = *a*·*x* + *b*,

with *a* and *b* being the slope and the intercept of the linear regression, respectively. In addition to these two characteristic values the associated coefficients of determination *R*^2^ are listed in the Table 1. A detailed overview of all calculated electrochemical potentials obtained at the different levels of theory in comparison to the experimental reference data is provided in Tables S1–S11 (ESI†). Graphical representations of the absolute deviations in the calculated *vs.* experimental electrochemical potentials are depicted in Fig. S2 and S3 (ESI†).

**Table 1:** Fit parameters of linear regression applied to the experimentally determined electrochemical potentials plotted against the results obtained for the different theoretical calculation methods. Since the second reduction potential of 2-OH-AQ proved to be a systematic outlier in all cases, the fitting parameters for the full set and after elimination of 2-OH-AQ are listed

		AQ/AQ˙^−^	AQ/AQ^2−^	
		Full	Full	Excluding 2-OH-AQ
B3LYP+D3	*a*	1.09 ± 0.04	1.00 ± 0.1	1.05 ± 0.06
	*b*/mV	68 ± 29	61 ± 187	121 ± 81
	*R* ^2^	0.987	0.854	0.976
SCC DFTB/3ob	*a*	1.01 ± 0.03	0.80 ± 0.10	0.90 ± 0.05
	*b*/mV	20 ± 25	−130 ± 155	−90 ± 64
	*R* ^2^	0.989	0.865	0.979
SCC DFTB/mio	*a*	0.92 ± 0.04	0.70 ± 0.10	0.80 ± 0.06
	*b*/mV	−12 ± 33	−321 ± 171	−239 ± 79
	*R* ^2^	0.980	0.785	0.959
GFN2-xTB/GBSA	*a*	0.87 ± 0.05	0.80 ± 0.10	0.83 ± 0.08
	*b*/mV	21 ± 42	−101 ± 144	−106 ± 111
	*R* ^2^	0.970	0.886	0.937
GFN2-xTB/ALPB	*a*	0.80 ± 0.04	0.71 ± 0.08	0.73 ± 0.05
	*b*/mV	−58 ± 31	−302 ± 116	−296 ± 64
	*R* ^2^	0.980	0.894	0.970

In the case of OH-substituted AQ derivatives, all possible conformations (*i.e. syn*- and *anti*-orientation of the OH-substituents with respect to the carbonyl group) were considered in the calculations. As expected, the *syn*-conformers (*i.e.* the OH-group pointing towards the carbonyl group) proved to be the most stable conformers, displaying the lowest energy in the quantum chemical calculations. Also, the calculated electrochemical potentials showed the best agreement with respect to the experimental reference values in all cases (data not shown). For this reason only data for the chemically relevant *syn*-conformers are reported in this work and used in the following analysis.

Among all methods considered in this study the B3LYP+D3 DFT level displays the best agreement when compared to the experimental data, albeit at a substantially increased computational effort compared to the simpler SCC DFTB methods. Among the latter, the 3ob parametrization, focused especially on the description of organic and bioorganic systems, showing a better performance when compared to the results obtained *via* SCC DFTB/mio and GFN2-xTB, respectively.

More specifically, in the case of B3LYP+D3, the coefficients of determination *R*^2^ of 0.987 and 0.976 (without consideration of 2-OH-AQ), obtained in the linear fit for the first and second reduction step, imply very good correlations between the measured and calculated potential data. Nevertheless, small deviations from the ideal correlation of 1.0 were identified in the respective slopes amounting to 1.09 and 1.05, along with notable intercepts of 68 and 121 mV, respectively.

In the case of the different DFTB models, slightly smaller *R*^2^-values in the range from 0.970 to 0.980 have been observed for the AQ/AQ˙^−^ step except of the SCC DFTB/3ob parametrization, showing a near-ideal correlation with an *R*^2^-value of 0.989. This implies that the performance of the individual theoretical models cannot be discriminated solely based on the correlation of determination. Visual inspection of the associated correlation plots shown in Fig. 5 reveals, that the the SCC DFTB/3ob level delivers a virtually ideal correlation well on par to that of the B3LYP+D3 calculations. This is also reflected by the associated slope and intercept of the first reduction step given as 1.01 and 20 mV, which are in fact slightly improved over the DFT results. On the other hand, the correlations obtained for SCC DFTB/mio and GFN2-xTB display a notably decreased accuracy. Due to the comparably poor performance of the GFN2-xTB/ALPB setting to GFN2-xTB/GBSA, it has not been included in Fig. 5.

A similar trend in the performance of the DFTB-based approaches is observed in the case of the second reduction step (AQ/AQ^2−^), albeit without the consideration of 2-OH-AQ, which proved to be a systematic outlier for all levels of theory considered in this study. The SCC DFTB/3ob level again displays the best performance being on par with that observed in the B3LYP+D3 case, except for the slope which amounts only to 0.90. However, in the case of all other methods the agreement with the experimental reference set is greatly diminished, albeit for different reasons. In the case of the GFN2-xTB/ALPB setting (see Fig. S1, ESI†) the largest deviation in the slope yielding a value of only 0.73 is observed, whereas the associated *R*^2^-value of 0.970 appears acceptable. Similarly, GFN2-xTB/GBSA displays the smallest *R*^2^-value of 0.937, whereas the largest magnitude in the intercept of −239 mV is observed in the case of SCC DFTB/mio.

In summary, the calculations at B3LYP+D3 and SCC DFTB/3ob levels were able to provide adequate data for the electrochemical potentials of different AQ derivatives when compared to the experimental reference. However, as mentioned above the second reduction step of 2-OH-AQ proved to be methodical outliers for all tested theoretical methods. As already noted above, the experimental CV graph shows notable deviations from the expected electrochemical response, indicating the occurrence of overpotentials in the re-oxidation reactions which can for instance be induced by kinetic limitations as well as side reactions such as dimerization. The latter can of course not be adequately represented in the theoretical calculations considering just a single target molecule.

This implies that promising candidate structures derived *via* virtual screening methods still have to be validated *via* experimental means. Considering the varying performance of the individual theoretical methods, a further analysis investigating the correlation between the individual theoretical models has been carried out.

In addition, a comparison of the experimentally obtained results in the absence of an implicit solvation model was performed for the different methods considered in this study. The corresponding comparison can be found in Table S13 and Fig. S8 (ESI†). While the associated *R*^2^ values in the case of the vacuum treatment indicate a good correlation between the experimental and calculated data, the strongly deviating slopes *a* and the large intercepts *b* clearly show that an omission of solvation effects in the calculation of the electrochemical potentials leads to highly inadequate results.

### 3.3 Correlation between theoretical methods

**Fig. 6:**
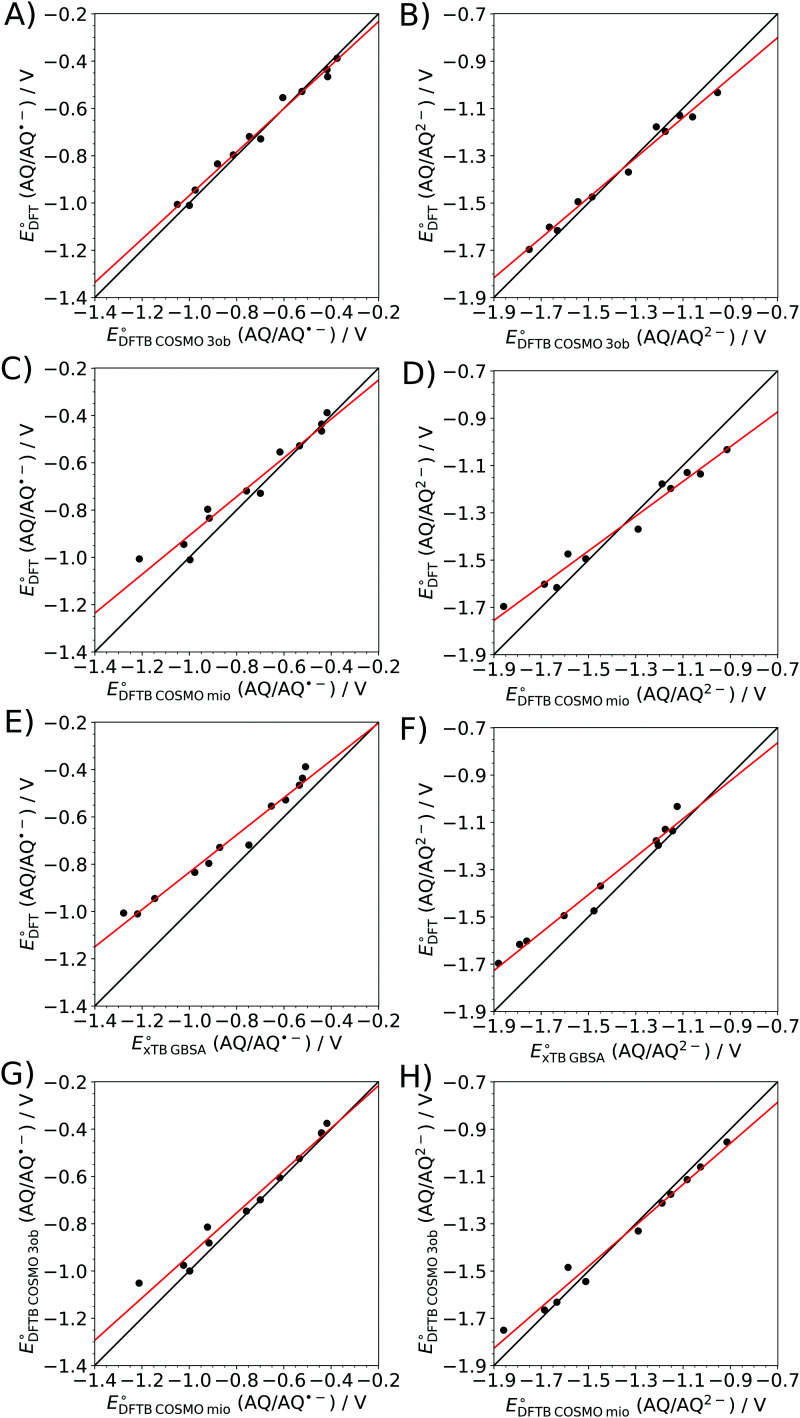
Correlation of the electrochemical potentials obtained *via* different theoretical models for the first (left) and second (right) reduction step of the considered AQ-derivatives determined *via* (A and B) SCC DFTB/3ob, (C and D) SCC DFTB/mio and (E and F) GFN2-xTB/GBSA against those obtained at the B3LYP+D3/6-31++G(d,p) level and (G and F) SCC DFTB/mio against SCC DFTB/3ob. Black line: ideal correlation. Red line: linear regression.

The varying qualities observed in the correlations between the calculated and experimental data prompted a comparison of the theoretical methods among each other. The associated correlation plots for the first and second reduction step are shown in Fig. 6, and the parameters obtained from the respective linear regressions are listed in Table 2. Also, in this case, graphical depictions of the absolute deviations between the calculated electrochemical potentials, obtained at different levels of theory, have been compiled (see the ESI,† Fig. S4–S7).

**Table 2:** Fit parameters of the linear regression applied to the electrochemical potentials obtained for individual pairs of theoretical methods

		AQ/AQ˙^−^	AQ/AQ^2−^
B3LYP+D3 *vs.* SCC DFTB/3ob	*a*	0.92 ± 0.04	0.85 ± 0.03
	*b*/mV	−49 ± 26	−209 ± 47
	*R* ^2^	0.986	0.986
B3LYP+D3 *vs.* SCC DFTB/mio	*a*	0.82 ± 0.06	0.73 ± 0.04
	*b*/mV	−86 ± 48	−360 ± 58
	*R* ^2^	0.950	0.972
B3LYP+D3 *vs.* GFN2-xTB/GBSA	*a*	0.79 ± 0.04	0.80 ± 0.04
	*b*/mV	−46 ± 36	−202 ± 66
	*R* ^2^	0.974	0.972
B3LYP+D3 *vs.* GFN2-xTB/ALPB	*a*	0.72 ± 0.04	0.70 ± 0.02
	*b*/mV	−122 ± 35	−393 ± 33
	*R* ^2^	0.970	0.990
SCC DFTB/mio *vs.* SCC DFTB/3ob	*a*	1.08 ± 0.06	1.13 ± 0.05
	*b*/mV	20 ± 43	180 ± 70
	*R* ^2^	0.973	0.983
GFN2-xTB/ALPB *vs.* GFN2-xTB/GBSA	*a*	1.09 ± 0.03	1.15 ± 0.04
	*b*/mV	99 ± 30	280 ± 59
	*R* ^2^	0.990	0.989

The most interesting comparison in this aspect is the benchmarking of the B3LYP+D3 calculations against the results obtained using the different DFTB and *x*TB approaches, since the latter show a substantially reduced computational demand compared to high level DFT calculations. In particular, the SCC DFTB/3ob level displays an exceptionally good correlation when compared to the B3LYP+D3 results (see Fig. 6A and B). The associated coefficients of determination *R*^2^ are 0.986 for both the one- and two-fold reduction steps. Even though the respective slopes of 0.92 and 0.85, and the comparably large intercept values of −49 mV and −209 mV, indicate a systematic deviation between the two methods, the obtained electrochemical potential data can still be regarded as highly correlated.

On the other hand, the correlations between the SCC DFTB/mio and B3LYP+D3 show notably higher deviations (see Fig. 6C and D). The regression parameters for the first reduction step, found as 0.82, −86 mV and 0.950 for the slope, intercept and *R*^2^-values are comparable to the SCC DFTB/3ob level. In contrast, the parameters determined for the second reduction step indicate large systematic deviations with the slope and intercept being 0.73 and −360 mV, albeit still maintaining an acceptable *R*^2^-value of 0.972. This decrease in accuracy, between the first and second reduction step, is most likely the result of the lower level in the methodical hierarchy of the SCC DFTB/mio approach, since it does not take third-order terms based on the respective Hubbard derivatives^66^ into account. The latter can be interpreted as a change of the ionic radii upon variation in the atomic occupations, and their neglect can be expected to result in more pronounced deviations upon increasing charges, compared to the DFT reference calculations and the 3ob parametrization, which explicitly accounts for these effects *via* third-order terms.

This conclusion is supported when correlating the two DFTB approaches against each other (see Fig. 6G and H). Indeed, the values determined for the slope, intercept and coefficient of determination of 1.08, 20 mV and 0.973 indicate a very good correlation between the 3ob and mio parametrization for the first reduction step. However, when considering the AQ/AQ^2−^ reaction a notable decline in the quality of the correlation is observed, especially in terms of the slope and intercept, yielding values of 1.13 and 180 mV, respectively. Nevertheless, the *R*^2^-value of 0.960 implies that despite these systematic offsets, the two methods still display a high level of correlation also in the case of the second reduction reaction. This finding is in good agreement with the conclusions drawn from the comparison between theory and experiment discussed above, in which the SCC DFTB/mio approach performs well for the first reduction reaction, while a notable decrease in the quality of the calculated values is observed upon twofold reduction.

The comparison observed in the case of the GFN2-xTB approach, in combination with either the ALPB and GBSA solvation model against the B3LYP+D3, reference leads to mixed conclusions. While on the one hand the *R*^2^-values remain within the range of 0.970 to 0.990, the respective slopes in the range of 0.72 to 0.80 point towards increased systematic deviations compared to the SCC DFTB methods. This is insofar surprising as the GFN2-xTB methods were designed to incorporate a number of improvements over third-order DFTB methods.^75–78^ However, as also observed in the case of the SCC DFTB/3ob approach the slope and *R*^2^-value did not suffer as much when changing from the first to the second reduction step when employing the GFN2-xTB approach. Similar to that in comparison with the experimental data the GBSA solvation model provides a better description compared to its ALPB counterpart. Interestingly, the correlation between the GFN2-xTB/ALPB and GFN2-xTB/GBSA yields near ideal *R*^2^-values of 0.990 and 0.989 in the case of the first and second reduction steps. The respective slopes of 1.09 and 1.15 and intercept values of 99 and 280 mV point towards notable systematic deviations between the employed solvation models. Thus, the reduced accuracy observed for both tested GFN2-xTB levels might in fact be linked to the performance of the employed solvation models, which could be inferior to the COSMO model employed in the SCC DFTB case.

One particularly surprising aspect in this comparison is the fact, that the second reduction step of the 2-OH-AQ molecule does not stand out as an outlier in any of the correlations between the considered theoretical methods. This implies that, despite the varying performance, a consistent theoretical prediction of the two-fold reduction in the case of 2-OH-AQ is observed, pointing towards a potential systematic deviation in the experimental measurement such as dimeriziation side reactions as already discussed above.

### 3.4 Molecular properties derived from DFT data

Based on the DFT calculation results characteristic data for the neutral AQ derivatives, namely the ionization potential, electron affinity, electronegativity and hardness have been determined and correlated with the experimentally determined electrochemical potential (see the ESI,† Fig. S9). In particular, the electron affinity, being proportional to the LUMO energy, shows a very good correlation with the experimental data. In contrast, the IP shows a surprisingly low correlation, and since the electronegativity and hardness are both derived from IP and EA data, the respective correlation against the experimental data is considerably poor as well.

In addition molecular data visualizations for the electrostatic surface potentials^26,82^ have been prepared for all AQ derivatives and correlated against the measured electrochemical potential as shown in Fig. S10 (ESI†).

## 4 Conclusion

In this work the impact of the number and placement of OH- and NH_2_-substituents on the experimentally determined electrochemical potentials of AQ and 12 AQ derivatives has been investigated and correlated against theoretically calculated values employing a variety of theoretical methods. With the exception of 2-OH-AQ, which demonstrates a diminished electrochemical response, most likely induced by side reactions such as dimerization, the CV plots of all systems displayed the expected features of two separate, one-electron reduction peaks with associated re-oxidation reactions. The expected mesomeric effects, associated with NH_2_-substitution, are well reflected in the respective potential shifts when compared to the unsubstituted AQ lead structure. An opposite trend was observed upon OH-substitution in agreement with the previous literature, which can be linked to the formation of intramolecular hydrogen bonds for hydroxy groups in the β-position.

The systematic evaluation of the quality of theoretically determined electrochemical potentials of the considered AQ derivatives, obtained *via* an isodesmic reaction approach against newly measured CV data, provides manifold insight into potential strategies for a virtual screening of these key compounds associated with applications in organic electronics. When considering the data obtained at the DFT level the successful work on benzo-, naphto- and anthraquinones reported by other research groups^39–42^ could be directly extended with high accuracy to a new set of AQ-based systems. By assessing the performance of various DFTB-based calculation methods, the SCC DFTB/3ob level in conjunction with the COSMO model proved to be a viable, low-demand alternative to the considerably more expensive B3LYP+D3/SMD approach. While the quality in correlation against the experimental reference data is slightly inferior to that of the employed DFT approach, the highly advantageous cost-accuracy ratio of the SCC DFTB/3ob/COSMO framework enables the formulation of a hierarchical screening strategy, in which the low-cost method can be efficiently employed in a coarse pre-screening protocol. Afterwards, the best candidate structures can be employed as an input for the higher level DFT treatment.

The comparison of the calculated electrochemical potentials obtained at different levels of theory also provides detailed insight into the inner workings of the employed calculation methods, directly highlighting the shortcomings of the respective approaches, *e.g.* the performance of second- *vs.* third-order DFTB methods. Moreover, the behaviour of the special case 2-OH-AQ, which did not show up as an outlier in comparisons of different theoretical approaches, enabled potentially challenging systems in the experimental measurements to be pinpointed. The deviations observed in the respective CV plots show the occurrence of overpotentials most likely induced by kinetic limitations or/and side reactions, thus making such compounds less attractive in electrochemical applications.

In addition to providing a systematic benchmark of the theoretically determined electrochemical potentials against newly measured experimental data, the outlined calculation strategy enables the formulation of a detailed workflow to set up and execute virtual screening studies of various organic compounds relevant for applications in sustainable materials science.

## Conflicts of interest

There are no conflicts to declare.

## Supplementary Material

CP-024-D2CP01717B-s001
