# Supplementary material for: Anthraquinone and its derivatives as sustainable materials for electrochemical applications – a joint experimental and theoretical investigation of the redox potential in solution
Source: Phys Chem Chem Phys. 2022 Jun 1;24(26):16207–19. doi: 10.1039/d2cp01717b (PMC9258729; doi:10.1039/d2cp01717b)
Supplement: CP-024-D2CP01717B-s001 [file CP-024-D2CP01717B-s001.pdf]

Electronic Supplementary Material (ESI) for Physical Chemistry Chemical Physics.
This journal is © the Owner Societies 2022

Anthraquinone and its Derivatives as Sustainable Materials for Electrochemical Applications – a Joint Experimental and Theoretical Investigation of the Redox Potential in Solution

Supplementary Material

Josef M. Gallmetzer[a], *Stefanie Kröll*[a], *Daniel Werner*[b], *Dominik Wielend*[c], *Mihai Irimia-Vladu*[c], *Engelbert Portenkirchner*[b,*,b], *Niyazi Serdar Sariciftci*[c], *Thomas S. Hofer*[a†]

[a]Theoretical Chemistry Division
Institute of General, Inorganic and Theoretical Chemistry
Center for Chemistry and Biomedicine
University of Innsbruck, Innrain 80-82, A-6020 Innsbruck, Austria

[b]Institute of Physical Chemistry
Josef-Möller-Haus
University of Innsbruck, Innrain 52c, A-6020 Innsbruck, Austria

[c]Linz Institute for Organic Solar Cells (LIOS)
Institute of Physical Chemistry
Johannes Kepler University Linz, Altenberger Strasse 69,
4040 Linz, Austria

June 20, 2022

*Corresponding author (experimental): engelbert.portenkirchner@uibk.ac.at
†Corresponding author (theory): t.hofer@uibk.ac.at

Table S1: Experimentally determined reduction potentials.

**Compound:**	$\Delta E^{\circ}(\mathrm{AQ/AQ^{\bullet -}})$ / mV	$\Delta E^{\circ}(\mathrm{AQ/AQ^{2-}})$ / mV
**AQ**	-683.6	-1432.3
**1-OH**	-530.0	-1178.5
**2-OH**	-707.5	-1139.8
**1,2-OH**	-542.9	-1125.5
**1,4-OH**	-423.9	-1066.4
**1,5-OH**	-369.1	-893.5
**1,8-OH**	-396.4	-1107.0
**1-NH$_2$**	-816.4	-1516.9
**2-NH$_2$**	-875.9	-
**1,2-NH$_2$**	-950.6	-1551.2
**1,4-NH$_2$**	-983.4	-1541.8
**2,6-NH$_2$**	-1086.0	-1652.3
**1-NH$_2$-4-OH**	-710.4	-1328.9

Table S2: Comparison of the electrochemical potential obtained for the first reduction step ($AQ/AQ^{\bullet -}$) at B3LYP+D3/SMD level against the experimental reference.

**Compound**	ΔE°_{calc} / mV	ΔE°_{meas} / mV	$\Delta\Delta E^{\circ}$ / mV
**AQ***	-683.6*	-683.6	0.0
**1-OH**	-528.1	-530.0	-1.9
**2-OH**	-718.8	-707.5	11.3
**1,2-OH**	-554.3	-542.9	11.4
**1,4-OH**	-466.0	-423.9	42.1
**1,5-OH**	-387.9	-369.1	18.8
**1,8-OH**	-436.5	-396.4	40.1
**1-NH_2**	-796.4	-816.4	-20.0
**2-NH_2**	-834.6	-875.9	-41.3
**1,2-NH_2**	-945.0	-950.6	-5.6
**1,4-NH_2**	-1010.4	-983.4	26.9
**2,6-NH_2**	-1006.3	-1086.0	-79.7
**1-OH-4-NH_2**	-728.8	-710.4	18.4

*reference value

Table S3: Comparison of the electrochemical potential obtained for the second reduction step (AQ/AQ^{2-}) at B3LYP+D3/SMD level against the experimental reference.

**Compound**	ΔE°_{calc} / mV	ΔE°_{meas} / mV	$\Delta\Delta E^{\circ}$ / mV
**AQ**	-1432.3*	-1432.3	0.0
**1-OH**	-1196.9	-1178.5	18.4
**2-OH**	-1474.4	-1139.8	**334.6**
**1,2-OH**	-1177.9	-1125.5	52.4
**1,4-OH**	-1135.9	-1066.4	69.5
**1,5-OH**	-1032.8	-893.5	**139.3**
**1,8-OH**	-1129.61	-1107.0	22.6
**1-NH_2**	-1494.4	-1516.9	-22.5
**1,2-NH_2**	-1601.9	-1551.2	50.8
**1,4-NH_2**	-1616.2	-1541.8	74.4
**2,6-NH_2**	-1696.3	-1652.3	44.0
**1-OH-4-NH_2**	-1369.2	-1328.9	40.3

*reference value

Table S4: Comparison of the electrochemical potential obtained for the first reduction step (AQ/AQ$^{\bullet-}$) at SCC DFTB/3ob/COSMO level against the experimental reference.

**Compound**	ΔE°_{calc} / mV	ΔE°_{meas} / mV	$\Delta\Delta E^{\circ}$ / mV
**AQ***	-683.6*	-683.6	0.0
**1-OH**	-524.1	-530.0	-5.9
**2-OH**	-746.7	-707.5	39.2
**1,2-OH**	-605.0	-542.9	62.1
**1,4-OH**	-415.5	-423.9	-8.4
**1,5-OH**	-375.5	-369.1	6.4
**1,8-OH**	-418.1	-396.4	21.7
**1-NH$_2$**	-814.4	-816.4	-2.0
**2-NH$_2$**	-881.3	-875.9	5.0
**1,2-NH$_2$**	-967.0	-950.6	25.4
**1,4-NH$_2$**	-1000.1	-983.4	16.6
**2,6-NH$_2$**	-1051.4	-1086.0	-34.6
**1-OH-4-NH$_2$**	-698.9	-710.4	-11.5

*reference value

Table S5: Comparison of the electrochemical potential obtained for the second reduction step (AQ/AQ^{2-}) at SCC DFTB/3ob/COSMO level against the experimental reference.

**Compound**	ΔE°_{calc} / mV	ΔE°_{meas} / mV	$\Delta\Delta E^{\circ}$ / mV
**AQ**	-1432.3*	-1432.3	0.0
**1-OH**	-1175.2	-1178.5	-3.3
**2-OH**	-1484.5	-1139.8	**344.7**
**1,2-OH**	-1212.9	-1125.5	87.3
**1,4-OH**	-1059.6	-1066.4	-6.8
**1,5-OH**	-954.3	-893.5	60.8
**1,8-OH**	-1113.5	-1107.0	6.54
**1-NH$_2$**	-1544.3	-1516.9	27.4
**1,2-NH$_2$**	-1665.2	-1551.2	114.0
**1,4-NH$_2$**	-1631.6	-1541.8	89.8
**2,6-NH$_2$**	-1750.5	-1652.3	98.2
**1-OH-4-NH$_2$**	-1330.9	-1328.9	2.0

*reference value

Table S6: Comparison of the electrochemical potential obtained for the first reduction step ($AQ/AQ^{\bullet-}$) at SCC DFTB/mio/COSMO level against the experimental reference.

**Compound**	ΔE°_{calc} / mV	ΔE°_{meas} / mV	$\Delta\Delta E^{\circ}$ / mV
**AQ***	-683.6*	-683.6	0.0
**1-OH**	-534.4	-530.0	4.4
**2-OH**	-758.3	-707.5	50.7
**1,2-OH**	-617.2	-542.9	74.3
**1,4-OH**	-440.6	-423.9	16.7
**1,5-OH**	-417.7	-369.1	48.6
**1,8-OH**	-440.5	-396.4	44.1
**1-NH$_2$**	-922.9	-816.4	106.5
**2-NH$_2$**	-916.1	-875.9	40.2
**1,2-NH$_2$**	-1023.8	-950.6	73.2
**1,4-NH$_2$**	-997.7	-983.4	14.3
**2,6-NH$_2$**	-1212.0	-1086.0	126.0
**1-OH-4-NH$_2$**	-699.48	-710.4	-10.9

*reference value

Table S7: Comparison of the electrochemical potential obtained for the second reduction step (AQ/AQ^{2-}) at SCC DFTB/mio/COSMO level against the experimental reference.

**Compound**	ΔE°_{calc} / mV	ΔE°_{meas} / mV	$\Delta\Delta E^{\circ}$ / mV
**AQ**	-1432.3*	-1432.3	0.0
**1-OH**	-1152.3	-1178.5	-26.2
**2-OH**	-1586.8	-1139.8	**447.0**
**1,2-OH**	-1188.9	-1125.5	-63.3
**1,4-OH**	-1026.2	-1066.4	-40.2
**1,5-OH**	-914.4	-893.5	20.9
**1,8-OH**	-1082.7	-1107.0	-24.3
**1-NH$_2$**	-1511.7	-1516.9	-5.2
**1,2-NH$_2$**	-1686.1	-1551.2	134.9
**1,4-NH$_2$**	-1634.1	-1541.8	92.3
**2,6-NH$_2$**	-1859.0	-1652.3	206.7
**1-OH-4-NH$_2$**	-1289.8	-1328.9	-39.08

*reference value

Table S8: Comparison of the electrochemical potential obtained for the first reduction step ($AQ/AQ^{\bullet -}$) at GFN2-xTB/GBSA level against the experimental reference.

**Compound**	ΔE°_{calc} / mV	ΔE°_{meas} / mV	$\Delta\Delta E^{\circ}$ / mV
**AQ***	-683.6*	-683.6	0.0
**1-OH**	-592.9	-530.0	62.3
**2-OH**	-748.5	-707.5	41.0
**1,2-OH**	-653.3	-542.9	110.4
**1,4-OH**	-535.1	-423.9	111.2
**1,5-OH**	-509.1	-369.1	140.0
**1,8-OH**	-521.8	-396.4	125.4
**1-NH$_2$**	-918.1	-816.4	101.7
**2-NH$_2$**	-977.3	-875.9	101.4
**1,2-NH$_2$**	-1147.0	-950.6	196.4
**1,4-NH$_2$**	-1219.5	-983.4	236.1
**2,6-NH$_2$**	-1278.3	-1086.0	192.3
**1-OH-4-NH$_2$**	-871.8	-710.4	161.4

*reference value

Table S9: Comparison of the electrochemical potential obtained for the second reduction step (AQ/AQ^{2-}) at GFN2-xTB/GBSA level against the experimental reference.

**Compound**	ΔE°_{calc} / mV	ΔE°_{meas} / mV	$\Delta\Delta E^{\circ}$ / mV
**AQ**	-1432.3*	-1432.3	0.0
**1-OH**	-1204.8	-1178.5	26.3
**2-OH**	-1476.4	-1139.8	**336.6**
**1,2-OH**	-1213.4	-1125.5	87.9
**1,4-OH**	-1143.9	-1066.4	77.5
**1,5-OH**	-1124.6	-893.5	231.1
**1,8-OH**	-1175.4	-1107.0	68.4
**1-NH$_2$**	-1603.1	-1516.9	86.2
**1,2-NH$_2$**	-1761.3	-1551.2	210.1
**1,4-NH$_2$**	-1791.1	-1541.8	249.3
**2,6-NH$_2$**	-1880.5	-1652.3	228.2
**1-OH-4-NH$_2$**	-1449.0	-1328.9	120.1

*reference value

Table S10: Comparison of the electrochemical potential obtained for the first reduction step ($AQ/AQ^{\bullet -}$) at GFN2-xTB/ALPB level against the experimental reference.

**Compound**	ΔE°_{calc} / mV	ΔE°_{meas} / mV	$\Delta\Delta E^{\circ}$ / mV
**AQ***	-683.6*	-683.6	0.0
**1-OH**	-548.7	-530.0	18.7
**2-OH**	-739.0.	-707.5	31.5
**1,2-OH**	-588.9	-542.9	46.0
**1,4-OH**	-467.6	-423.9	43.7
**1,5-OH**	-451.6	-369.1	82.5
**1,8-OH**	-478.6	-396.4	82.2
**1-NH$_2$**	-888.2	-816.4	71.8
**2-NH$_2$**	-1029.9	-875.9	154.0
**1,2-NH$_2$**	-1100.3	-950.6	149.8
**1,4-NH$_2$**	-1200.9	-983.4	217.5
**2,6-NH$_2$**	-1317.8	-1086.0	231.8
**1-OH-4-NH$_2$**	-822.1	-710.4	111.7

*reference value

Table S11: Comparison of the electrochemical potential obtained for the second reduction step (AQ/AQ^{2-}) at GFN2-xTB/ALPB level against the experimental reference.

**Compound**	ΔE°_{calc} / mV	ΔE°_{meas} / mV	$\Delta\Delta E^{\circ}$ / mV
**AQ**	-1432.3*	-1432.3	0.0
**1-OH**	-1145.5	-1178.5	-33.0
**2-OH**	-1464.7	-1139.8	**324.9**
**1,2-OH**	-1124.7	-1125.5	-0.8
**1,4-OH**	-1035.9	-1066.4	-30.5
**1,5-OH**	-944.0	-893.5	50.5
**1,8-OH**	-1076.7	-1107.0	-30.3
**1-NH_2**	-1579.6	-1516.9	62.7
**1,2-NH_2**	-1747.6	-1551.2	196.4
**1,4-NH_2**	-1727.9	-1541.8	186.1
**2,6-NH_2**	-1891.4	-1652.3	239.1
**1-OH-4-NH_2**	-1371.7	-1328.9	42.8

*reference value

Table S12: Isodesmic reaction: Calculation of derivative reduction potentials using a reference reaction.

X-AQ	+	e^-	$\longrightarrow$	$\text{X-AQ}^{\bullet -}$			$-FE^\circ$
$\text{AQ}^{\bullet -}$			$\longrightarrow$	AQ	+	e^-	FE°_{ref}
$\text{AQ}^{\bullet -}$	+	X-AQ	$\longrightarrow$	AQ	+	$\text{X-AQ}^{\bullet -}$	ΔG°_r

Table S13: Fit parameters of the linear regression applied to the experimentally determined electrochemical potentials plotted against the results obtained for the different theoretical calculation methods in vacuum.

		AQ / AQ$^{\bullet-}$	AQ / AQ^{2-}	
		full	full	excluding 2-OH-AQ
B3LYP+D3	a	0.84 ± 0.02	0.78 ± 0.12	0.86 ± 0.03
	b / mV	-145 ± 16	-320 ± 150	-250 ± 34
	R^2	0.992	0.826	0.992
SCC DFTB/3ob	a	0.78 ± 0.04	0.62 ± 0.10	0.69 ± 0.04
	b / mV	-198 ± 29	-536 ± 124	-486 ± 44
	R^2	0.974	0.812	0.979
SCC DFTB/mio	a	0.76 ± 0.03	0.60 ± 0.10	0.66 ± 0.04
	b / mV	-179 ± 19	-537 ± 171	-495 ± 48
	R^2	0.989	0.818	0.974
GFN2-xTB	a	0.77 ± 0.02	0.69 ± 0.09	0.73 ± 0.03
	b / mV	-156 ± 13	-426 ± 113	-403 ± 38
	R^2	0.995	0.873	0.987

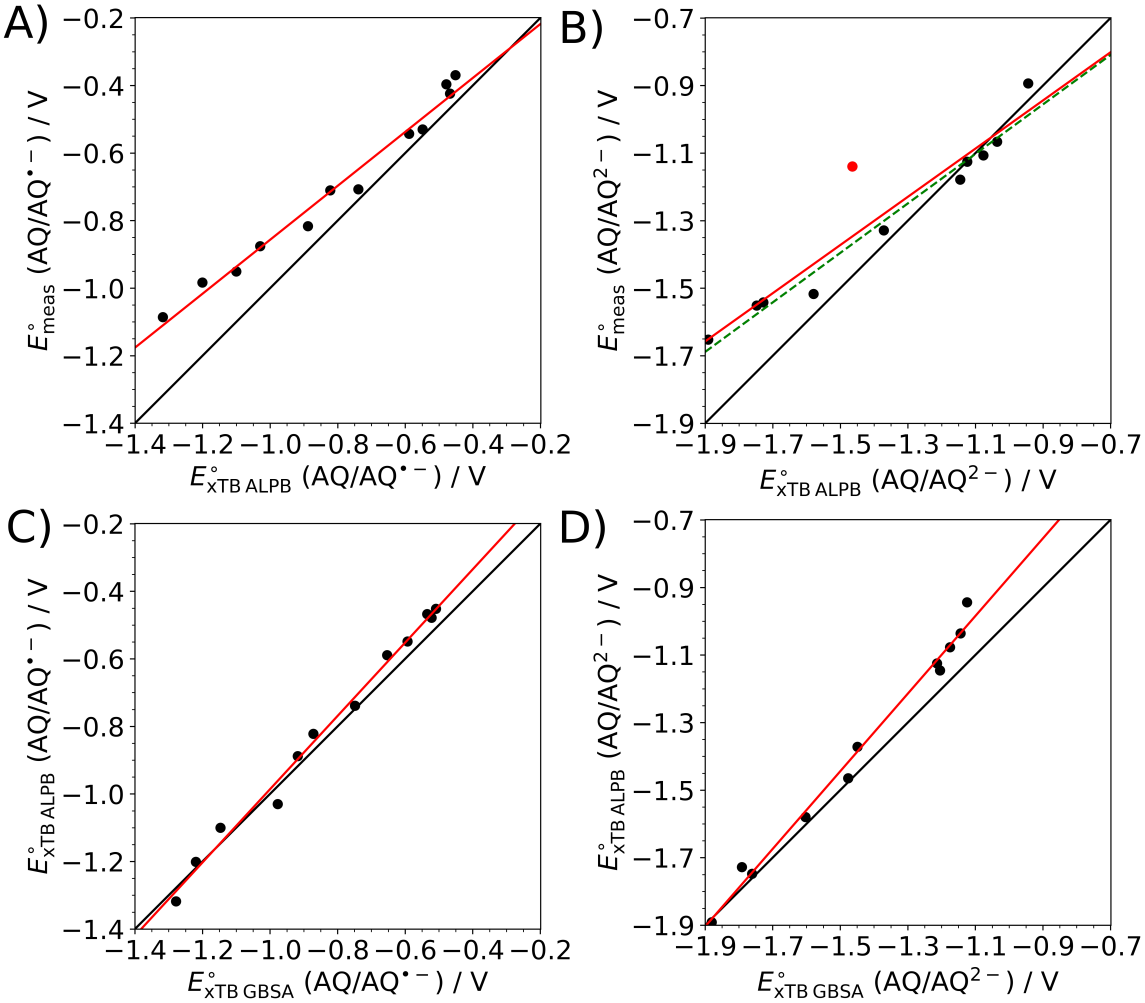

Figure S1: Correlation between the electrochemical potentials for the first (left) and second (right) reduction step of different AQ-derivatives between A,B) the experimental reference and the GFN2-xTB/ALPB level as well as C,D) the GFN2-xTB/ALPB and GFN2-xTB/GBSA levels, respectively. Black line: ideal correlation. Red line: linear regression. Green dashed line: linear regression without the outlier 2-OH-AQ marked by a red dot.

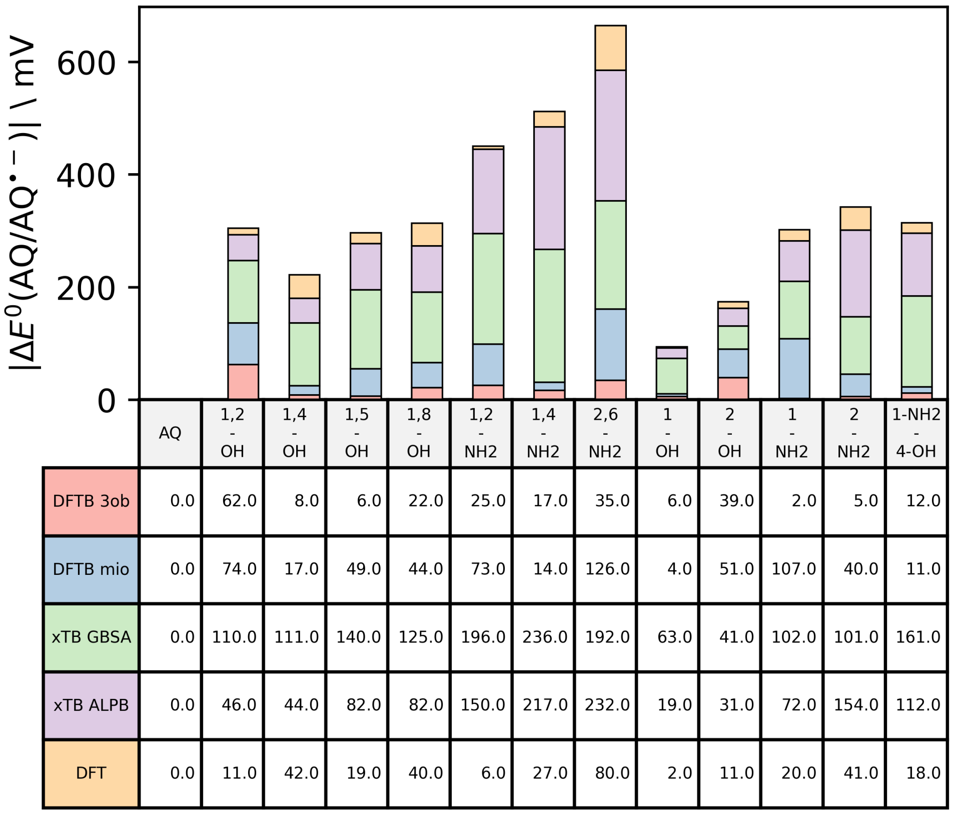

	AQ	1,2-OH	1,4-OH	1,5-OH	1,8-OH	1,2-NH2	1,4-NH2	2,6-NH2	1-OH	2-OH	1-NH2	2-NH2	1-NH2-4-OH
DFTB 3ob	0.0	62.0	8.0	6.0	22.0	25.0	17.0	35.0	6.0	39.0	2.0	5.0	12.0
DFTB mio	0.0	74.0	17.0	49.0	44.0	73.0	14.0	126.0	4.0	51.0	107.0	40.0	11.0
xTB GBSA	0.0	110.0	111.0	140.0	125.0	196.0	236.0	192.0	63.0	41.0	102.0	101.0	161.0
xTB ALPB	0.0	46.0	44.0	82.0	82.0	150.0	217.0	232.0	19.0	31.0	72.0	154.0	112.0
DFT	0.0	11.0	42.0	19.0	40.0	6.0	27.0	80.0	2.0	11.0	20.0	41.0	18.0

Figure S2: Comparison of the absolute deviation between the employed computational methods from the experimental reference for the first reduction step ($AQ/AQ^{\bullet-}$).

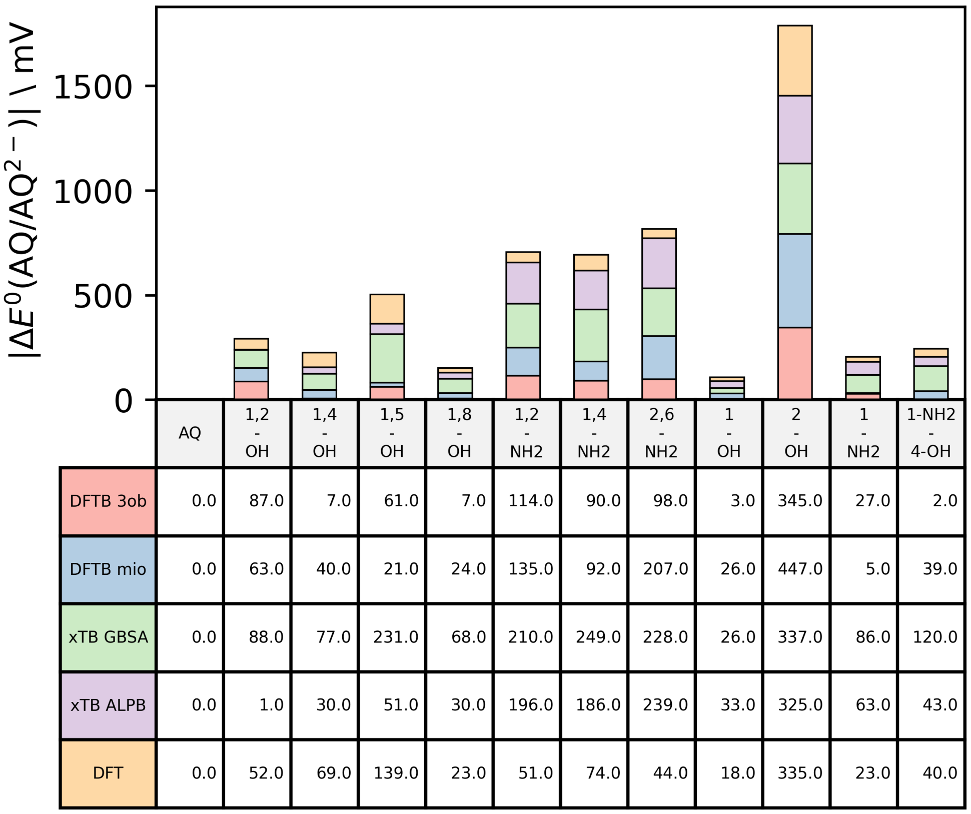

	AQ	1,2 - OH	1,4 - OH	1,5 - OH	1,8 - OH	1,2 - NH2	1,4 - NH2	2,6 - NH2	1 - OH	2 - OH	1 - NH2	1-NH2 - 4-OH
DFTB 3ob	0.0	87.0	7.0	61.0	7.0	114.0	90.0	98.0	3.0	345.0	27.0	2.0
DFTB mio	0.0	63.0	40.0	21.0	24.0	135.0	92.0	207.0	26.0	447.0	5.0	39.0
xTB GBSA	0.0	88.0	77.0	231.0	68.0	210.0	249.0	228.0	26.0	337.0	86.0	120.0
xTB ALPB	0.0	1.0	30.0	51.0	30.0	196.0	186.0	239.0	33.0	325.0	63.0	43.0
DFT	0.0	52.0	69.0	139.0	23.0	51.0	74.0	44.0	18.0	335.0	23.0	40.0

Figure S3: Comparison of the absolute deviation between the employed computational methods from the experimental reference for the first reduction step (AQ/AQ^{2-}).

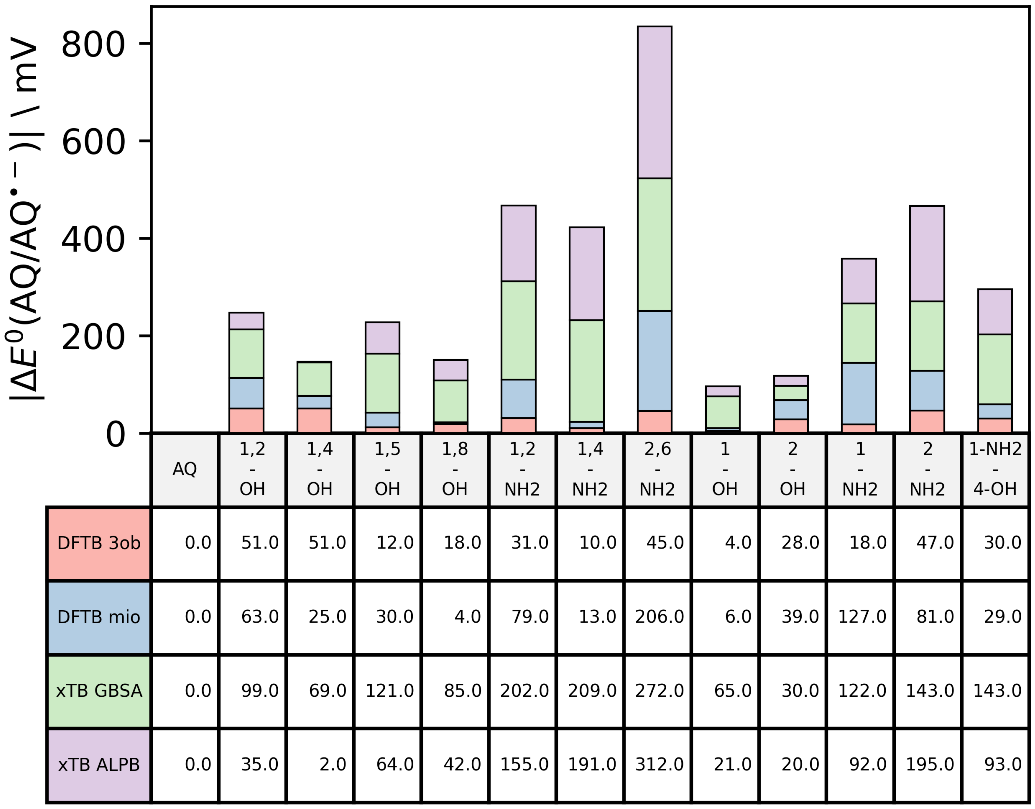

	AQ	1,2 - OH	1,4 - OH	1,5 - OH	1,8 - OH	1,2 - NH2	1,4 - NH2	2,6 - NH2	1 - OH	2 - OH	1 - NH2	2 - NH2	1-NH2 - 4-OH
DFTB 3ob	0.0	51.0	51.0	12.0	18.0	31.0	10.0	45.0	4.0	28.0	18.0	47.0	30.0
DFTB mio	0.0	63.0	25.0	30.0	4.0	79.0	13.0	206.0	6.0	39.0	127.0	81.0	29.0
xTB GBSA	0.0	99.0	69.0	121.0	85.0	202.0	209.0	272.0	65.0	30.0	122.0	143.0	143.0
xTB ALPB	0.0	35.0	2.0	64.0	42.0	155.0	191.0	312.0	21.0	20.0	92.0	195.0	93.0

Figure S4: Comparison of the absolute deviation between the employed DFTB and xTB methods from the B3LYP+D3 reference for the first reduction step ($AQ/AQ^{\bullet -}$).

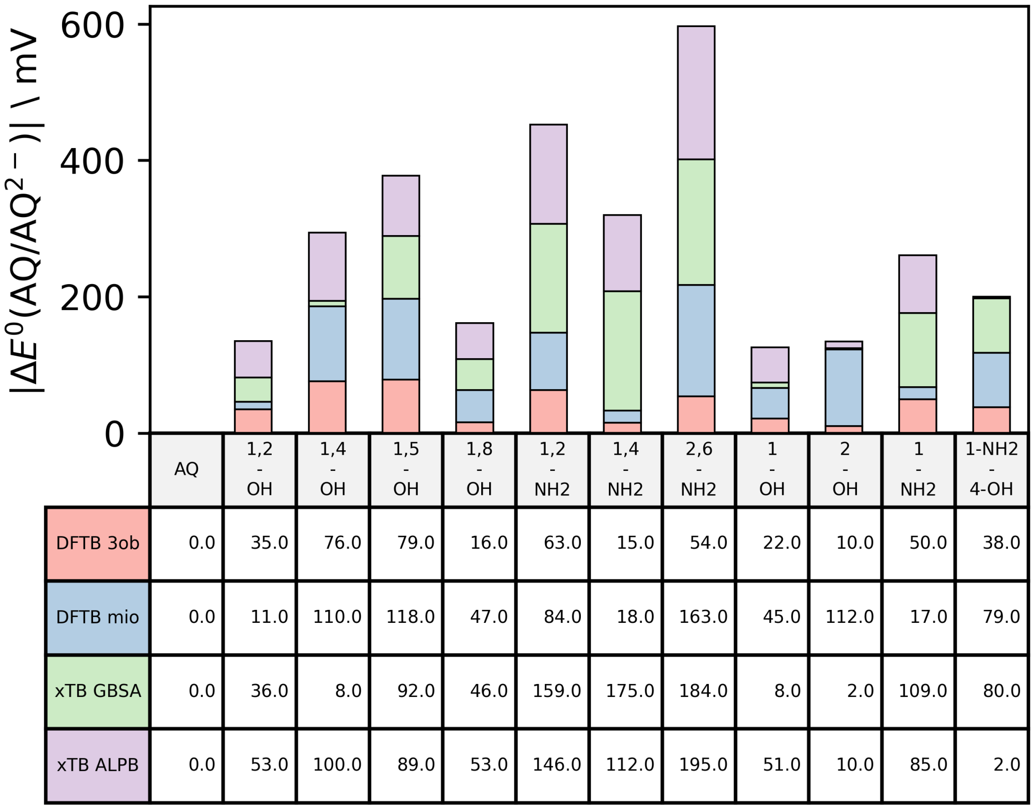

	AQ	1,2 - OH	1,4 - OH	1,5 - OH	1,8 - OH	1,2 - NH2	1,4 - NH2	2,6 - NH2	1 - OH	2 - OH	1 - NH2	1-NH2 - 4-OH
DFTB 3ob	0.0	35.0	76.0	79.0	16.0	63.0	15.0	54.0	22.0	10.0	50.0	38.0
DFTB mio	0.0	11.0	110.0	118.0	47.0	84.0	18.0	163.0	45.0	112.0	17.0	79.0
xTB GBSA	0.0	36.0	8.0	92.0	46.0	159.0	175.0	184.0	8.0	2.0	109.0	80.0
xTB ALPB	0.0	53.0	100.0	89.0	53.0	146.0	112.0	195.0	51.0	10.0	85.0	2.0

Figure S5: Comparison of the absolute deviation between the employed DFTB and xTB methods from the B3LYP+D3 reference for the second reduction step (AQ/AQ^{2-}).

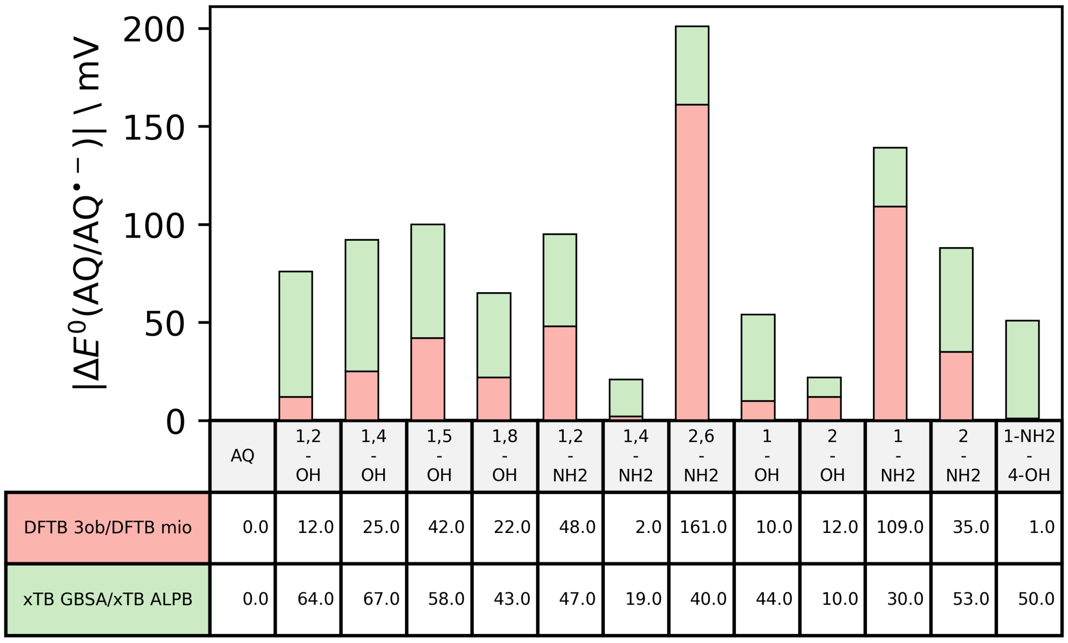

	AQ	1,2-OH	1,4-OH	1,5-OH	1,8-OH	1,2-NH2	1,4-NH2	2,6-NH2	1-OH	2-OH	1-NH2	2-NH2	1-NH2-4-OH
DFTB 3ob/DFTB mio	0.0	12.0	25.0	42.0	22.0	48.0	2.0	161.0	10.0	12.0	109.0	35.0	1.0
xTB GBSA/xTB ALPB	0.0	64.0	67.0	58.0	43.0	47.0	19.0	40.0	44.0	10.0	30.0	53.0	50.0

Figure S6: Comparison of the absolute deviation between the SCC DFTB/3ob and SCC DFTB/mio levels as well as between GFN2-xTB/ALPB and GFN2-xTB/GBSA for the first reduction step ($AQ/AQ^{\bullet -}$) of the different AQ derivatives.

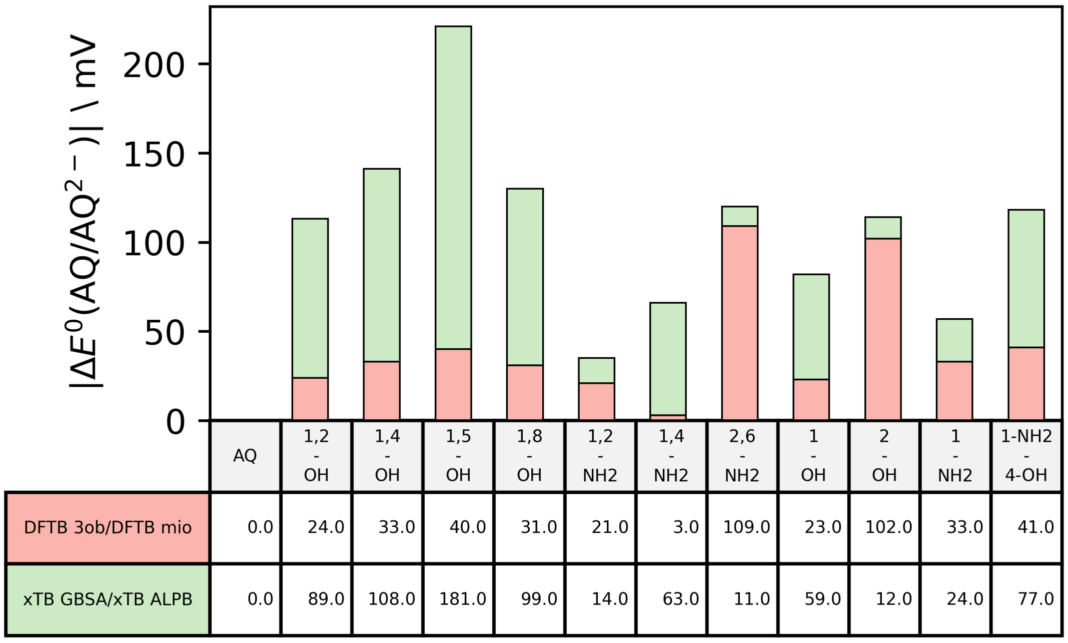

	AQ	1,2 - OH	1,4 - OH	1,5 - OH	1,8 - OH	1,2 - NH2	1,4 - NH2	2,6 - NH2	1 - OH	2 - OH	1 - NH2	1-NH2 - 4-OH
DFTB 3ob/DFTB mio	0.0	24.0	33.0	40.0	31.0	21.0	3.0	109.0	23.0	102.0	33.0	41.0
xTB GBSA/xTB ALPB	0.0	89.0	108.0	181.0	99.0	14.0	63.0	11.0	59.0	12.0	24.0	77.0

Figure S7: Comparison of the absolute deviation between the SCC DFTB/3ob and SCC DFTB/mio levels as well as between GFN2-xTB/ALPB and GFN2-xTB/GBSA for the second reduction step (AQ/AQ^{2-}) of the different AQ derivatives.

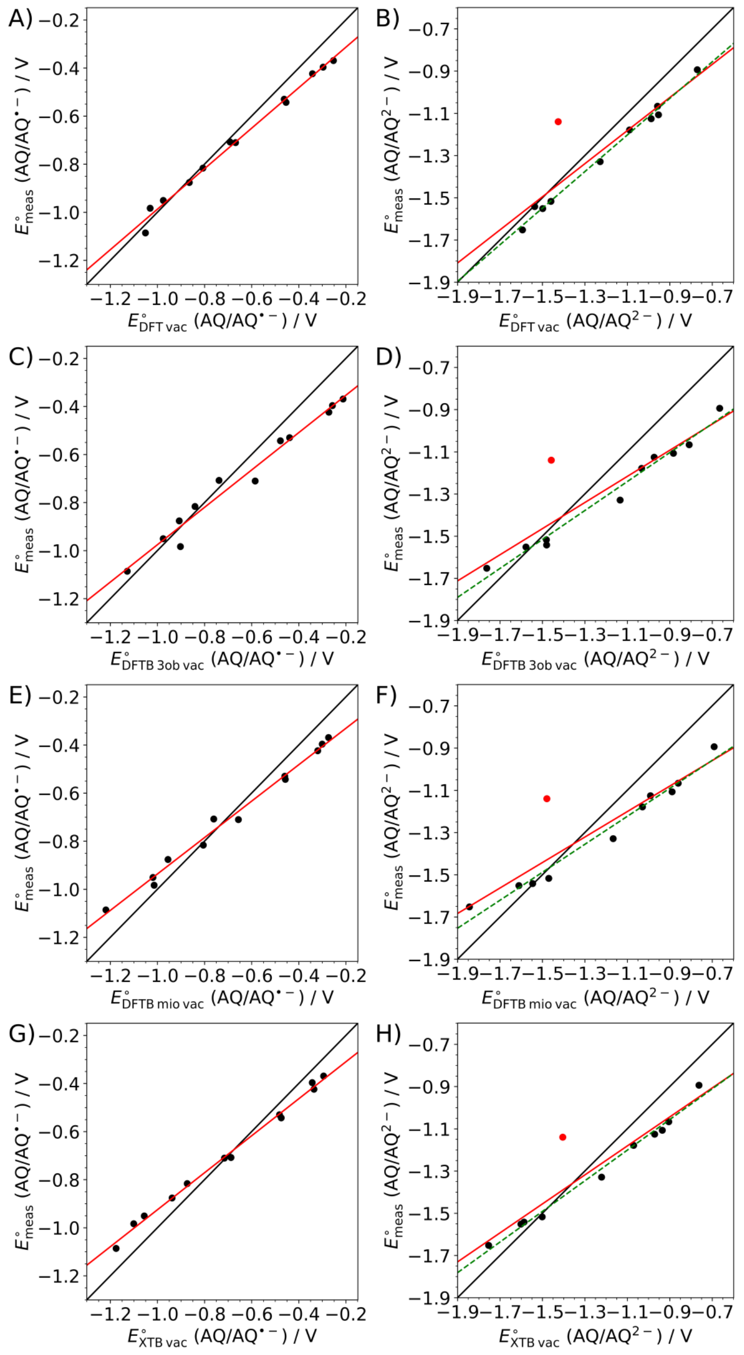

Figure S8: Correlation between the experimentally and theoretically determined electrochemical potentials for the first (left) and second (right) reduction step of the considered AQ-derivatives obtained at A,B) B3LYP+D3/6-31++G(d,p), C,D) SCC DFTB/3ob, E,F) SCC DFTB/mio and G,H) GFN2-xTB level in vacuum. Black line: ideal correlation. Red line: linear regression. Green dashed line: linear regression without the outlier 2-OH-AQ marked by a red dot.

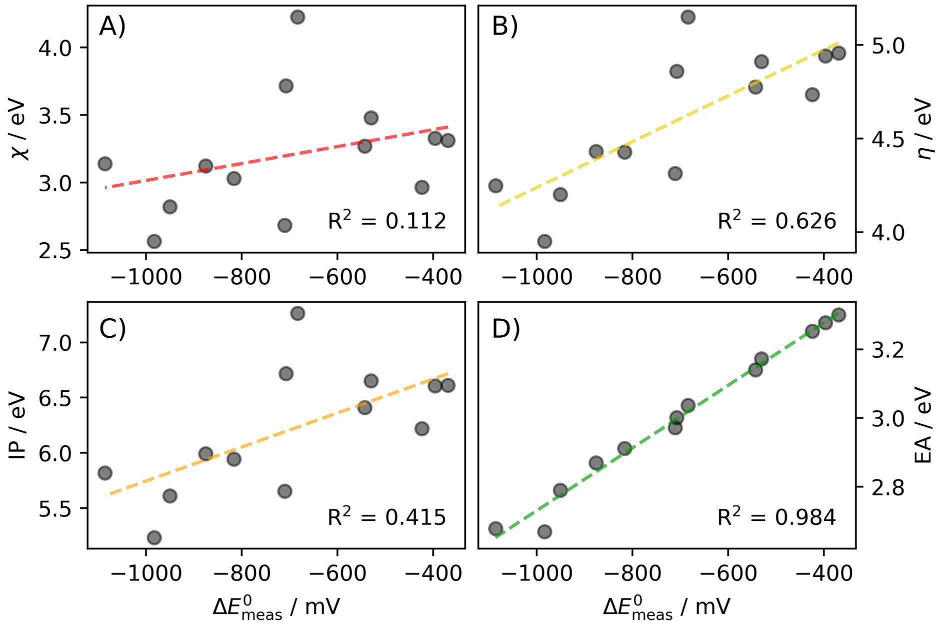

Figure S9: Correlation between the experimentally measured first reduction potential and A) the electronegativity χ, B) hardness η, C) ionisation potential IP, D) electron affinity EA, calculated with B3LYP+D3/6-31++G(d,p).

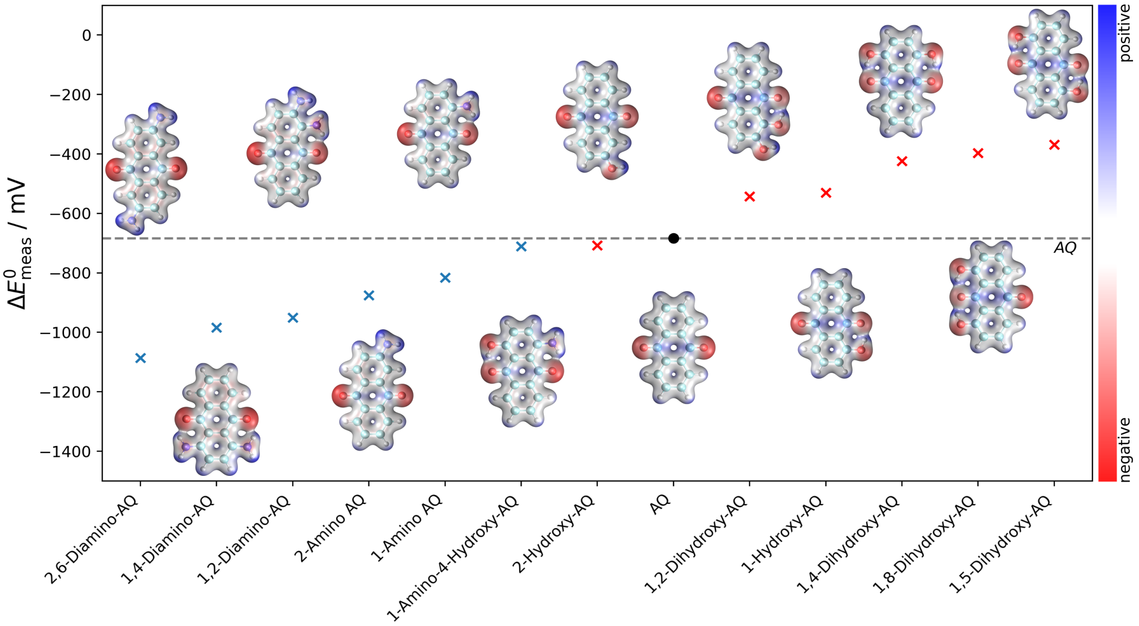

Figure S10: Representation of the experimentally measured first reduction potential with electrostatic potential projected on an isodensity of the associated electron density (isovalue = 0.025) calculated with B3LYP+D3/6-31++G(d,p).
